# Multiscale Embedded Gene Co-expression Network Analysis

**DOI:** 10.1371/journal.pcbi.1004574

**Published:** 2015-11-30

**Authors:** Won-Min Song, Bin Zhang

**Affiliations:** Department of Genetics and Genomic Sciences, Icahn Institute of Genomics and Multiscale Biology, Icahn School of Medicine at Mount Sinai, New York, New York, United States of America; National Research Council of Canada, CANADA

## Abstract

Gene co-expression network analysis has been shown effective in identifying functional co-expressed gene modules associated with complex human diseases. However, existing techniques to construct co-expression networks require some critical prior information such as predefined number of clusters, numerical thresholds for defining co-expression/interaction, or do not naturally reproduce the hallmarks of complex systems such as the scale-free degree distribution of small-worldness. Previously, a graph filtering technique called Planar Maximally Filtered Graph (PMFG) has been applied to many real-world data sets such as financial stock prices and gene expression to extract meaningful and relevant interactions. However, PMFG is not suitable for large-scale genomic data due to several drawbacks, such as the high computation complexity O(|V|^3^), the presence of false-positives due to the maximal planarity constraint, and the inadequacy of the clustering framework. Here, we developed a new co-expression network analysis framework called Multiscale Embedded Gene Co-expression Network Analysis (MEGENA) by: i) introducing quality control of co-expression similarities, ii) parallelizing embedded network construction, and iii) developing a novel clustering technique to identify multi-scale clustering structures in Planar Filtered Networks (PFNs). We applied MEGENA to a series of simulated data and the gene expression data in breast carcinoma and lung adenocarcinoma from The Cancer Genome Atlas (TCGA). MEGENA showed improved performance over well-established clustering methods and co-expression network construction approaches. MEGENA revealed not only meaningful multi-scale organizations of co-expressed gene clusters but also novel targets in breast carcinoma and lung adenocarcinoma.

## Author Summary

We developed a novel co-expression network analysis framework named Multiscale Embedded Gene co-Expression Network Analysis (MEGENA) that can effectively and efficiently construct and analyze large scale planar filtered co-expression networks. Two key components of MEGENA are the parallelization of embedded network construction and the identification of multi-scale clustering structures. MEGENA was applied to the breast cancer (BRCA) and the lung adenocarcinoma (LUAD) data from The Cancer Genome Atlas (TCGA) and showed much improved performance over well-established co-expression network approaches such as un-weighted and weighted gene co-expression network analyses. MEGENA revealed not only biologically meaningful multi-scale clustering structures of gene co-expression in both BRCA and LUAD, but also novel key regulators of important cancer biological processes like lineage-specific differentiations in LUAD. MEGENA is complementary to the established co-expression network analysis approaches by its capability of sparsifying densely connected co-expression networks and identifying multiscale modular structures.

## Introduction

Often, complex diseases involve multiple intertwined signaling circuitries. Cancer is an excellent example with a number of biological machineries activated in tumor pathogenesis including proliferation, angiogenesis, avoidance of cell death, evasion of tumor suppressing mechanisms, immortality, invasion etc[1]. The complexity of cancer further manifests via “tumor microenvironment”, a concept that incorporates interactions between not only the tumor cells, but also normal cells that contribute to the expression of the cancer hallmarks[2].

In many cases, networks of these intertwined signaling cascades, such as protein-protein interaction networks and metabolic networks are highly heterogeneous[3–5]. Particularly, these networks share certain characteristics such as the scale-free property (the degree distribution follows a power law), small world effect (diameter of network scales with logarithm/double-logarithm of the number of nodes)[3, 5], assortativity (preference for a network’s nodes to attach to others that are similar in some ways, i.e., high degree nodes tend to attach to high/low degree nodes)[6], and community structures [7, 8]. These observations suggest that the biological networks may follow the similar evolutionary dynamics, and thus network analysis approaches from other domains are very helpful for understanding biological networks[4].

These organizational principles are reflected in transcriptional control of cells: highly modular and yet diverse functional patterns emerge by means of “co-expression” [9, 10]. Co-expressed gene clusters represent coherent unique functional pathways not only in normal conditions [10, 11], but also in disease states[9, 12–14]. These “guilt-by-association” approaches were further extended to encapsulate gene-gene interactions by regarding genes as nodes and interactions as links, known as “co-expression network analysis”. These methods first evaluate the association strength between each gene pair by a similarity score (e.g., Pearson’s correlation coefficient) or statistical significance of the association, then identify co-expressed clusters or communities in the context of network topology[9, 15, 16].

However, the existing techniques to construct co-expression networks suffer from a number of drawbacks. For instance, some popular co-expression networks such as those from Weighted Gene Co-expression Network Analysis (WGCNA) enforce the connectivity to exhibit a power-law distribution[16], unweighted networks by hard thresholds contain a large number of false positive interactions[17], k-nearest-neighbor networks require the number of neighbors to connect by subjective criteria such as connectedness[15], and partial correlation based co-expression networks require at least O(|V|^3^) computational complexity[18], limiting the practical applications to |V| < 10^4^. These are further complicated by clustering analysis to identify modular organization of these networks. Some widely used clustering methods such as k-means and spectral clustering require predefined number of clusters[19].

More importantly, many of network-theoretic clustering methods are incapable of different levels of aggregations of clusters co-existing within a single network. There are several factors accounting for this particular drawback. Firstly, Newman’s modularity measure suffers the inherent resolution limit that fails to differentiate certain configurations of obvious clusters[20]. Secondly, they are often restricted to identify a single partition of a network by optimizing for the modularity, thus overlook multiscale organization of complex networks where coarse-grained and compact clusters co-exist[8].

In order to account for these shortcomings, we adopted a network embedding paradigm on a topological sphere. In other words, a co-expression network is embedded on a spherical surface such that one link does not cross the others. Planar Maximally Filtered Graph (PMFG) was developed to extract most relevant information from similarity matrices based on topological sphere, and has been applied mostly in financial domain[21]. PMFG becomes an ideal platform to construct co-expression networks due to the following attractive features: i) the preservation of hierarchy by retaining Minimum Spanning Tree (MST) as a subgraph, ii) the correspondence between a coherent cluster (if any) and a connected subnetwork, iii) the abundance of 3- and 4-cliques and exhibition of rich clustering structures[21], and iv) the possession of a wide spectrum of fundamental network characteristics in embedded networks such as transitions between scale-free to exponential degree distributions, and large-world to semi-ultra-small world[22, 23]. Applications to financial data have revealed that characteristic features of complex systems such as emergence of bubbles[24, 25], aggregation of similar firms in same sectors[24], highly connected hubs and hierarchical organizations[26, 27]. Furthermore, an embedded network inference framework called “Directed Bubble Hierarchical Tree” (DBHT)[27] was developed to infer meaningful clustering and hierarchical structures in PMFGs from gene expression, financial, and simulated data [27].

However, the existing PMFG embedding technique cannot efficiently handle large-scale genomic data. Firstly, pair-wise similarities are noisy and redundant, yielding high false-positive rates in identifying gene-gene interactions[17]. Enforcing maximal planarity inevitably introduces a significant number of these redundant links in a filtered network and may obscure the underlying “true” interactions. Secondly, the computation complexity for testing planarity is too high (*O*(|*V*|^*γ*^), 2 ≤ *γ* ≤ 3) for large scale network analysis. Thirdly, clustering analysis in PMFG via DBHT framework is not optimal. DBHT framework is based on inference of the patterns between separating triangles in PMFG, and requires that every node belongs to at least one triangle. Noting that gene-gene interactions do not necessarily form triangles, DBHT framework may not assign these genes to appropriate clusters. Lastly, a rigorous and formal definition of multiscale organization in these networks has been ignored. Although hierarchical structures have been exploited via agglomerative hierarchical clustering within bubbles and bubble clusters, they are inherently limited by aforementioned drawbacks of the bubble topology, and require a more rigorous algorithm to extract the full information encoded in embedded networks.

Here we developed a new network construction and analysis framework named Multiscale Embedded Gene Co-expression Network Analysis (MEGENA) to resolve the aforementioned issues with PMFG and DBHT, and more broadly with the existing co-expression network analysis methods. In the rest of the paper, we will briefly overview MEGENA, and then perform a comprehensive performance comparison of MEGENA and the established network construction and clustering analysis approaches using a series of simulated data as well as the real-world large-scale gene expression data. Finally, we will address the advantages of MEGENA and highlight some novel insights derived by MEGENA.

## Results

In this section, we first give an overview of MEGENA and address its algorithmic scalability. Then, we perform a comprehensive evaluation of MEGENA-derived Planar Filtered Networks (PFNs) and MEGENA-derived multi-scale clusters. We evaluate interactions captured by PFNs using a series of simulated and real-world gene expression data in comparison with False Discovery Rate (FDR) based networks (FDRN). We further compare the functional relevance of MEGENA-derived multi-scale clusters and those identified by several established clustering analysis approaches. Simulated expression data as well as the gene expression data in breast carcinoma (BRCA) and lung adenocarcinoma (LUAD) from The Cancer Genome Atlas (TCGA) were used. A detailed description about the data acquisition and preprocessing can be found in **S1 Text**. We proceed to highlight some novel insights revealed by multi-scale clusters and then conclude the paper by summarizing the key contributions and pointing out future work.

### Overview of MEGENA

**Fig 1:**
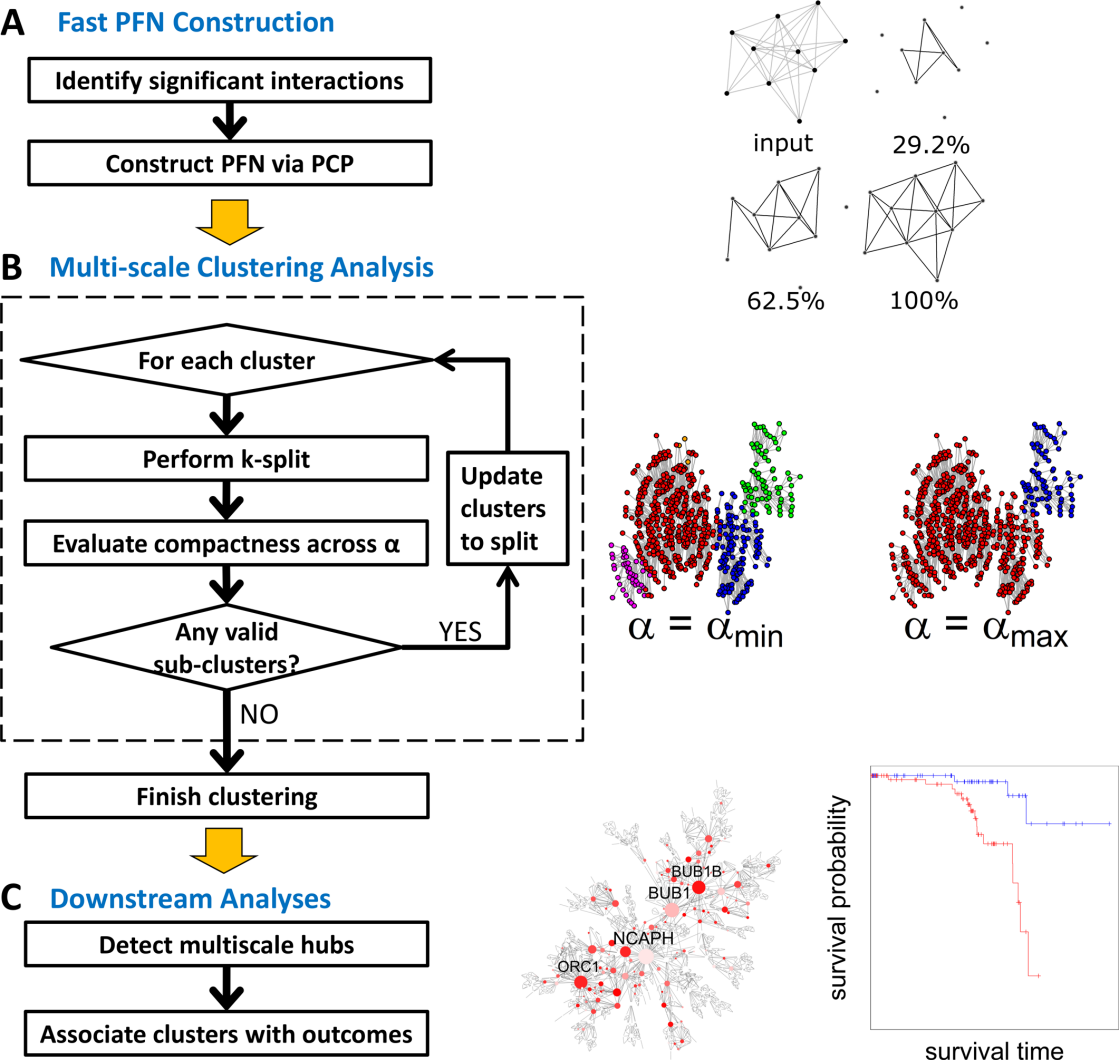
Flow chart of MEGENA. A) Fast planar filtered network construction. Significant interactions are first identified and then embedded on topological surface via a parallelized screening procedure described in the text. On the right, a toy example is illustrated to show construction of PFN from a thresholded network by FDR (top left), and gradual construction of PFN with number of included links and screened pairs shown on the top of each. B) Multi-scale clustering: Beginning from connected components of the initial PFN as the parent clusters, clustering is performed for each parent cluster and compactness of the sub-clusters are evaluated. These steps are described in the dotted box. The clustering is performed iteratively until there remains no further parent clusters meaningful to split. C) Downstream analyses: Multiscale Hub Analysis (MHA) is performed to detect significant hubs of individual clusters and across α, characterizing different scales of organizations in PFN. Then, clusters are ranked by associations to clinical traits including enrichment of differentially expressed gene (DEG) signatures, and correlations to survival end-point etc.

MEGENA consists of four major steps: **1)** Fast Planar Filtered Network construction (FPFNC) by introducing parallelization, early termination and prior quality control; **2)** Multiscale Clustering Analysis (MCA) by introducing compactness of modular structures characterized by a resolution parameter; **3)** Multiscale Hub Analysis (MHA) to identify highly connected hubs of each cluster at each scale and **4)** Cluster-Trait Association Analysis (CTA) to explore the relevance of cluster to clinical outcomes. **Fig 1** shows the overall analysis flow of MEGENA. Below we give a brief description of FPFNC, MCA and MHA. The details about these steps are presented in **Methods**.

FPFNC constructs PFN by mostly following the network embedding rationale from the PMFG algorithm. All pairs of genes are first ranked via a similarity measure quantifying respective interaction strengths and then iteratively tested for planarity to grow the embedded network that favors inclusion of pairs with larger similarities[21]. To make the PFN construction scalable for whole genome co-expression network analysis, two techniques were developed. Firstly, insignificant interactions are removed before the network embedding step by controlling the False Discovery Rate (FDR) of similarity for each gene pair. However, such a filtering may not be necessary since we will show in the subsequent section of **Evaluation of PFNs** that PFNs are very robust with respect to different FDR thresholds. Secondly, a parallelized screening procedure (PCP) is developed to extract a subset of gene pairs that are more likely to be embedded. Such procedures enable FPFNC to efficiently and effectively construct embedded co-expression networks by capturing significant interactions at the whole genome level.

PFN constructed through FPFNC is then input to MCA to identify multiscale clusters. MCA incorporates three distinct criteria to identify locally coherent clusters while maintaining a globally optimal partition. First, shortest path distances (SPD)[28] are utilized to optimize within-cluster compactness. Second, local path index (LPI) is used to optimize local clustering structure. Third, overall modularity (Q)[29] is employed to identify optimal partition. Specifically, MCA adopts a hierarchical divisive approach to dissect complex interactions in PFN into coherent interactomes across different resolution scales by iterating two steps, *k-split* and compactness evaluation. *k-split* identifies the clusters that lead to an optimal partition of a parent network via optimization of SPD, LPI and Q. In the step of compactness evaluation, individual clusters from *k-split* are compared to the parent network via a measure of network compactness defined below,

$$\upsilon =\frac{\bar{SPD}}{\log{\left(\left|V\right|\right)}^{\alpha }}$$ (1)

where, *V* is the set of nodes in the network, $\bar{SPD}$ is the average of shortest path distances of all node pairs, and *α* is the resolution parameter. Given that the denominator log(|*V*|)^*α*^ is the hallmark of the small-world property represented by the scaling relation $\hat{SPD}∼\log\left(|V|\right)$ when α = 1, *ν* measures the coherence of a network’s topology. Therefore, a smaller *α* identifies more compact clusters. For a given cluster (network), MCA searches through a range of α values for a resolution scale that leads to more compact clusters than the parent cluster (network). These clusters are further split by *k-split* until no more compact clusters can be identified. Each split represents a finer picture of modular structure of the given PFN. The output of MCA is a hierarchy of clusters at various levels defined by α.

Finally, MHA and CTA constitute the downstream analyses in MEGENA. MHA first identifies significant hubs within each cluster with respect to an established random model of planar networks[23, 29–31]. The nodes that are hubs at multiple scales are called multiscale hubs. CTA evaluates the relevance of individual clusters to clinical outcomes through principal component and correlation analyses.

### Scalability of MEGENA: Effectiveness of PCP

PCP is a key technique developed to speed up PFN construction to overcome the worst case O(|V|^3^) complexity of the existing serial PMFG algorithm (See **Methods** for detailed discussion) [21, 27]. In conjunction with correlation screening, PCP-mediated FPFNC dramatically increases its efficiency in construction whole genome co-expression network. In order to verify this, we compared PCP-mediated network embedding and the existing serial PMFG using the TCGA gene expression data that involve over 20,000 genes. We compared the acceptance rate of pairs filtered by PCP in MEGENA and that of non-filtered pairs by PMFG. The acceptance rate, defined as |E|/|E|_max_, where |E| is the number of edges embedded in a PFN, and |E|_max_ = 3(|V| - 2) is the maximal number of edges embeddable in a planar network by Euler relation [32].

**Fig 2:**
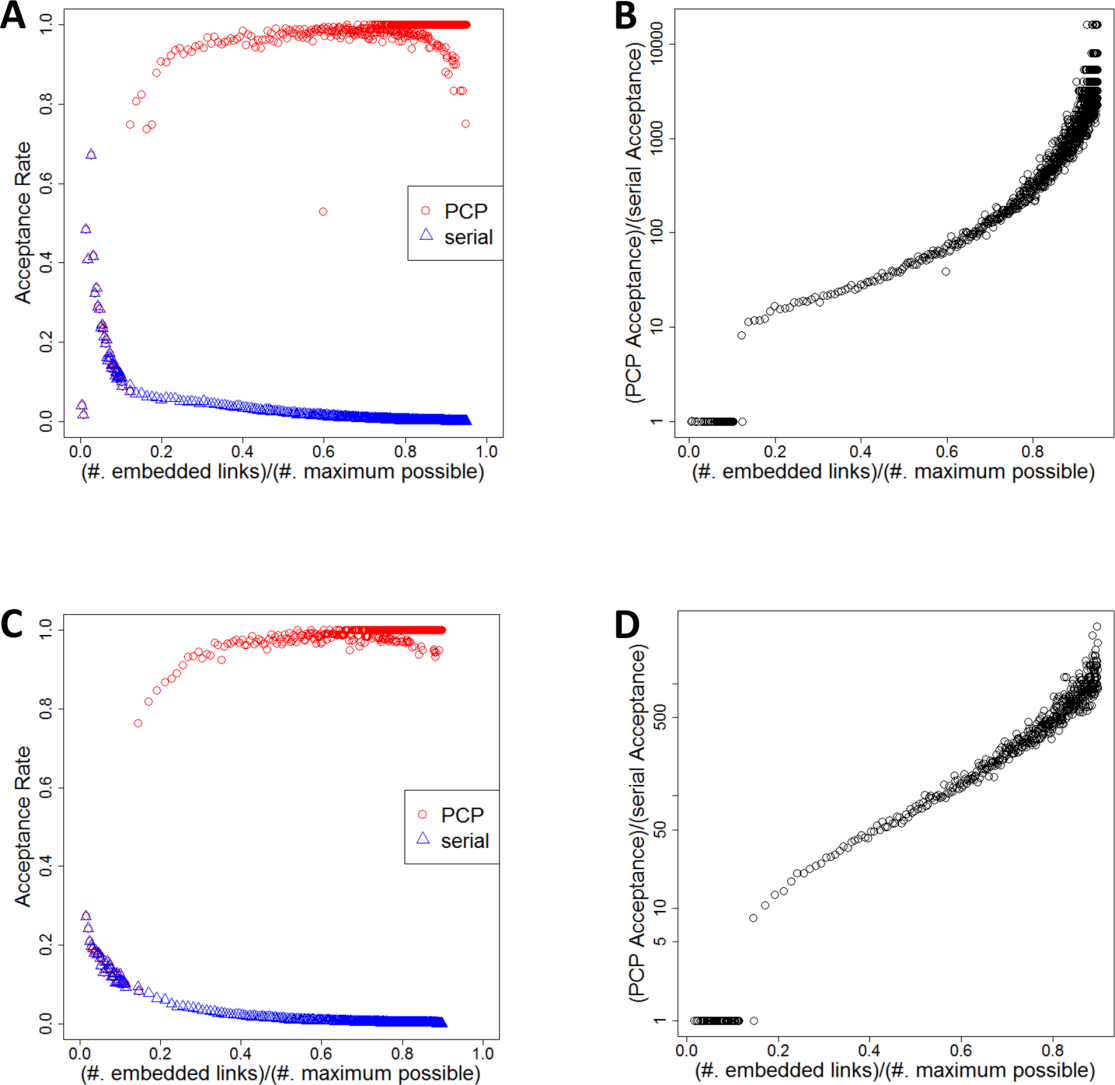
Comparison of acceptance rates of correlation pairs into PFN links. A,B) Results from PFN construction from TCGA lung squamous cell carcinoma (LUSC) data including 20523 genes. 57562 links out of maximal possible link number of 61563 are embedded. The left panel (A) shows the acceptance rates without PCP (denoted as “serial”, and colored as blue), and after performing PCP (denoted as “PCP”, and colored as red), as a function of number of links already embedded on the PFN, normalized by the maximum possible number of embedded links. The right panel (B) shows the ratio of acceptance rates after PCP to the acceptance rates without PCP is plotted as a function number of links already embedded on the PFN, normalized by the maximum possible number of embedded links. C,D) Results from TCGA thyroid carcinoma (THCA) data including 16639 genes. 44802 out of maximal possible link number of 49911 are embedded. The right and left panel show the same plots as described in the case of LUSC.

As shown in **Fig 2**, the acceptance rate by the serial PMFG algorithm quickly decreases close to 0% as the number of links in PFN reaches the maximal number of links. The finding indicates that PMFG performs exponentially increasing number of computations to embed more edges as the number of links in PFN saturates towards the maximal number. On the contrary, PCP remedies the problem by dramatically boosting the acceptance rate close to 100% as the number of links in PFN increases. These results demonstrate the effectiveness of PCP in reducing the overall computation time by leveraging parallel computation capability, and scalability of FPFNC for whole-genome co-expression network.

### Evaluation of PFNs

We evaluated the performance of PFNs from multiple aspects. We first evaluated capacity of PFNs in capturing underlying regulatory interactions by comparing to golden standard networks using simulated datasets from DREAM challenge[33]. We then compared the network neighborhoods of a number of genes in PFNs with their actual targets derived from the perturbation experiments. Furthermore, we compared the global topological properties of inferred PFNs with the established hallmark signatures of complex networks.

#### Evaluation by simulated data

To evaluate the accuracy of PFN in capturing the underlying gene regulatory network, we leveraged GeneNetWeaver[34] to generate 10 time series datasets for each of the golden standard networks from DREAM challenge[33]. The generation of the simulated data was detailed in **S1 Text**. We systematically compared MEGENA-derived PFNs with those based on the state-of-art methods including ARACNE[35] and Random Forest (RF)[36] across various FDR thresholds and similarity/dissimilarity measures. Specifically, we tested Pearson’s correlation coefficient (denoted as *PCC*), Mutual Information (denoted as *MI*), and Euclidean distance (denoted as *euclid*), and applied FDR thresholds (0.01, 0.05, 0.1, 0.2, 0.4, 0.6, 0.8 and 1) on each similarity/dissimilarity measure to filter out insignificant interactions. FDR thresholded interactions were then used to construct PFNs and ARACNE networks. Note that the three measures are all applicable to PFN, but ARACNE uses only MI as it assumes Data Processing Inequality (DPI) imposed by MI[35]. As RF is not dependent on any similarity/dissimilarity measure, FDR of the interactions determined by RF is estimated by permuting input data.

For each inferred network from a simulated data, we compared the weighted shortest path distances for all pairs of the nodes in the network and those in the underlying gold standard network. The node pairs connected in a gold standard network were treated as a positive class and the pairs not connected were taken as a negative class. We then calculated Area Under Curve in Receiver Operating Characteristic curve (AUC-ROC) for shortest path lengths in each inferred network.

**Fig 3:**
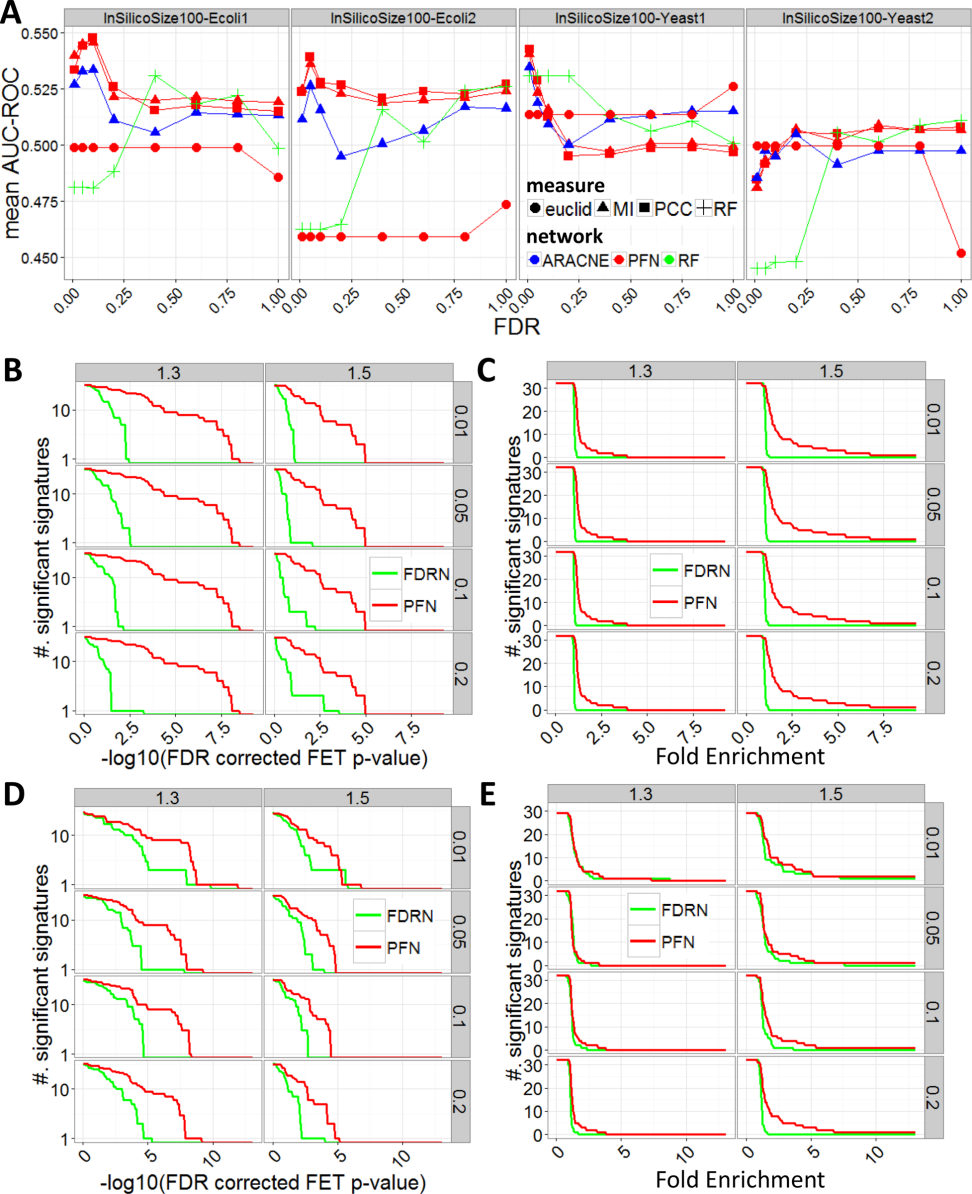
Validation of PFNs in comparison to various network inference methods. A. Comparisons of AUC of ROC for weighted shortest path distances of inferred networks from simulated data from various golden standard networks (labeled on the top), in comparison to ARACNE and RF. Different combinations with Pearson’s correlation coefficient (Pearson), mutual information (MI) and Euclidean distance (Euclid) were tested. B-C. Comparison of BRCA TF knock down signatures on BRCA PFN (red) and FDRN (green) neighborhoods of the target TFs, inferred from MI. The strips on the top of each plot shows expression fold changes (1.3 and 1.5 respectively) to derive these signatures. B shows FDR corrected FET p-values against the number of significantly enriched signatures. C shows enrichment fold change cut-off against the number of significantly enriched signatures. D-E. Comparisons of BRCA TF knock down signatures on inferred networks from PCC. D and E correspond to FDR corrected FET p-values and enrichment fold changes, similarly to B and C.

As shown in **Fig 3A**, the PFNs from PCC and MI consistently outperform the RF and ARACNE networks across various FDR thresholds. **Table 1** shows the best average AUC-ROC scores, indicating that PFNs from PCC and MI across various FDR thresholds show consistently the best performance except for InSilicoSize100-Yeast2 data set where PFNs are only slightly outperformed by RF based networks at an FDR threshold of 1. At FDR thresholds of 0.2 or less are practically used in almost all cases, PFNs from PCC and MI show the best overall performance.

**Table 1:** Table of best average AUC-ROC across various FDR thresholds. Each column represents the combination of network inference method and similarity/dissimilarity measure tested, and each row represents gold standard networks from which time series were generated. The best performing methods are highlighted by bold font.

Data id	ARACNE-MI	PFN-euclid	PFN-MI	PFN-PCC	RF
*InSilicoSize100-Ecoli1*	0.533405292	0.49876496	0.545384	**0.547641**	0.530597
*InSilicoSize100-Ecoli2*	0.526151567	0.47360654	0.535886	**0.539061**	0.525967
*InSilicoSize100-Yeast1*	0.534447108	0.52589657	0.540465	**0.542483**	0.530622
*InSilicoSize100-Yeast2*	0.504651933	0.49969052	0.508685	0.507814	**0.510899**

To assess the stability of the performance of each method, we calculated Coefficient of Variation (CV) of the average AUC-ROC scores across different FDR thresholds. PFNs from different similarity/dissimilarity measures have the most stable performance among the tested networks across different FDR thresholds ranging from 0 to 1 (**S1 Fig)**. The performance of PFNs peaks at FDR thresholds around 0.01 and/or 0.05 in most cases. However, PFNs from Euclidean distance have relatively poor performance in general.

#### Evaluation by disease-specific data

We further evaluated the accuracy of PFNs in detecting the true interactions in a disease dataset, we constructed PFN from BRCA gene expression data set from TCGA (hence, BRCA PFN). The details about the data acquisition and preprocessing can be found in **S1 Text**. For comparison with the BRCA PFN, we also constructed FDR thresholded network (FDRN) with FDR < 0.05 for PCC and MI. These co-expression networks were then tested for the enrichment of the siRNA knockdown signatures of key transcription factors (TFs) of breast carcinoma in MCF7 cells [37]. Of 78 TFs in the knockdown experiments, 32 TFs remained after data processing (see Data Acquisition and Processing in **S1 Text**), and appeared in the BRCA PFN and their siRNA signatures were used for testing the networks. Namely, the 32 TFs are: *BCL2*, *BRCA1*, *BRCA2*, *CCL5*, *CCNA2*, *CCNB1*, *CDC20*, *CDC25A*, *CDC25B*, *CDKN2A*, *CEBPB*, *CEBPD*, *CENPE*, *CENPF*, *CHEK1*, *E2F1*, *E2F5*, *ERBB2*, *ESR1*, *FOS*, *FOXC1*, *GATA3*, *HIF1A*, *HOXB7*, *ID1*, *MYBL2*, *MYC*, *PAX3*, *SKP2*, *STAT1*, *TOP2A*, and *WT1*. Two differentially expressed gene (DEG) signatures for each experiment were identified based on T-test p value < 0.05 and fold changes ≥ 1.3 or 1.5. Two different fold change cut-offs were chosen to give a more comprehensive performance.

For each co-expression network, we tested the enrichment of a TF’s DEG signature in its *l*-layer network neighborhoods (*l* = 1,…,*l*_max_, where *l*_max_ is the largest shortest distance between the given TF and the other nodes in the network). The optimal layer *l*_optimal_ was chosen based on FET p-value that shows the most significant enrichment of the DEG signature. The final FET p-values were adjusted for multiple testing. In the case of MI, **Fig 3B and 3C** show the number of signatures enriched in the BRCA PFN and FDRN at various thresholds for the corrected FET p-values and enrichment fold changes, respectively. **Fig 3D and 3E** show the results for the PCC based coexpression networks. Clearly, these experiment-derived gene signatures are far more significantly enriched in the PFNs than in the FDRNs, implying that the PFNs constructed by MEGENA can better capture true gene regulatory relationships using either PCC or MI.

#### Evaluation by topological characteristics

**Fig 4:**
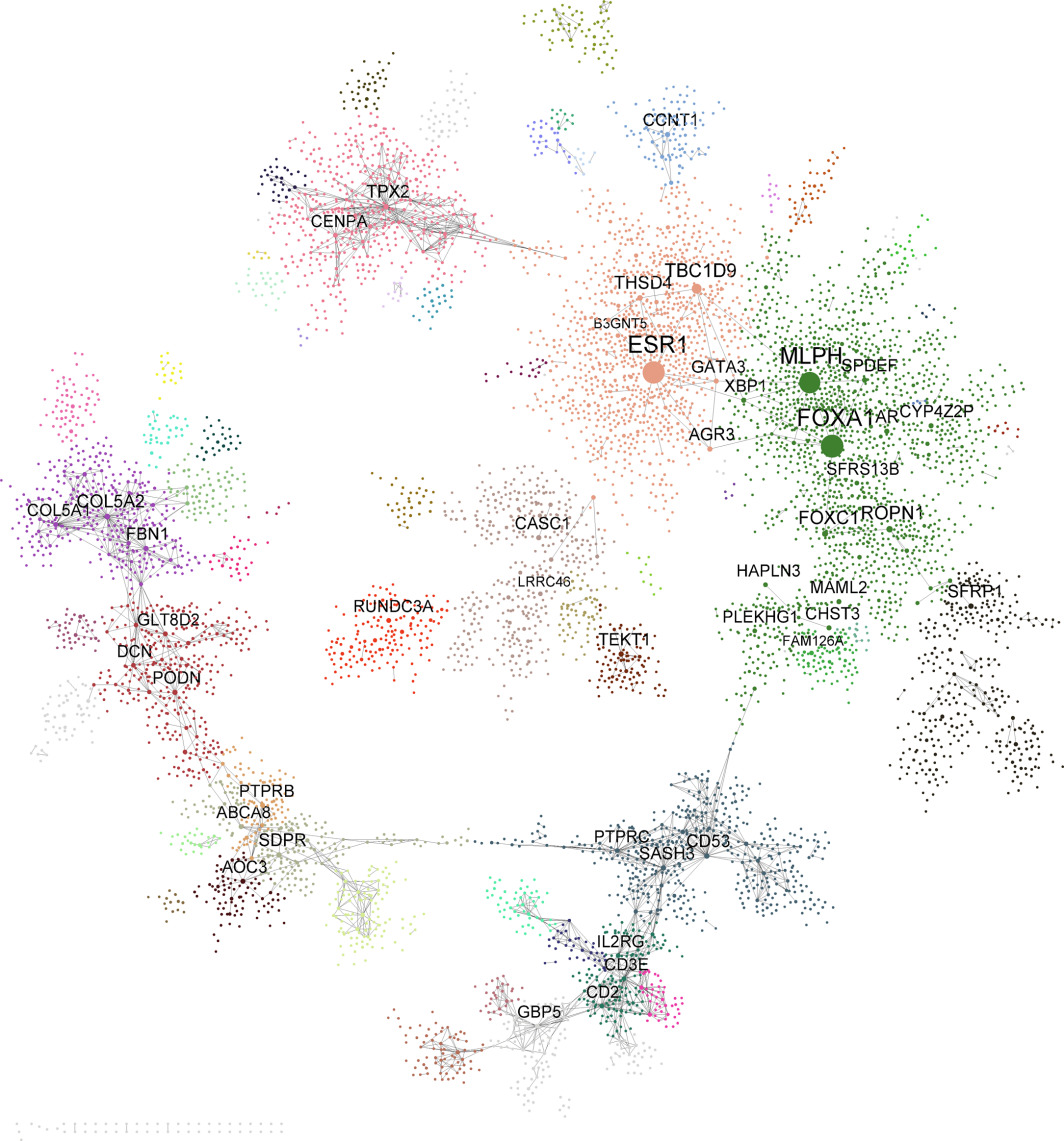
The global BRCA PFN. Different node colors represent different clusters identified at a scale of α = 1.3. Node size and label size are proportional to node degree.

Degree distributions and diameters of PFNs were computed since these are the key network topological characteristics representing the hallmark features such as scale-free and small-world networks [28]. We present the PFNs constructed from PCC in this section. **Fig 4** shows the global PCC-based BRCA PFN and its clusters identified at a scale defined by α = 1.3, and **S2 Fig** shows the global PCC-based LUAD and its clusters at α = 1. We reserve “k” to denote the node degree only in this section, but use k to denote the number of clusters in the other sections.

**Fig 5:**
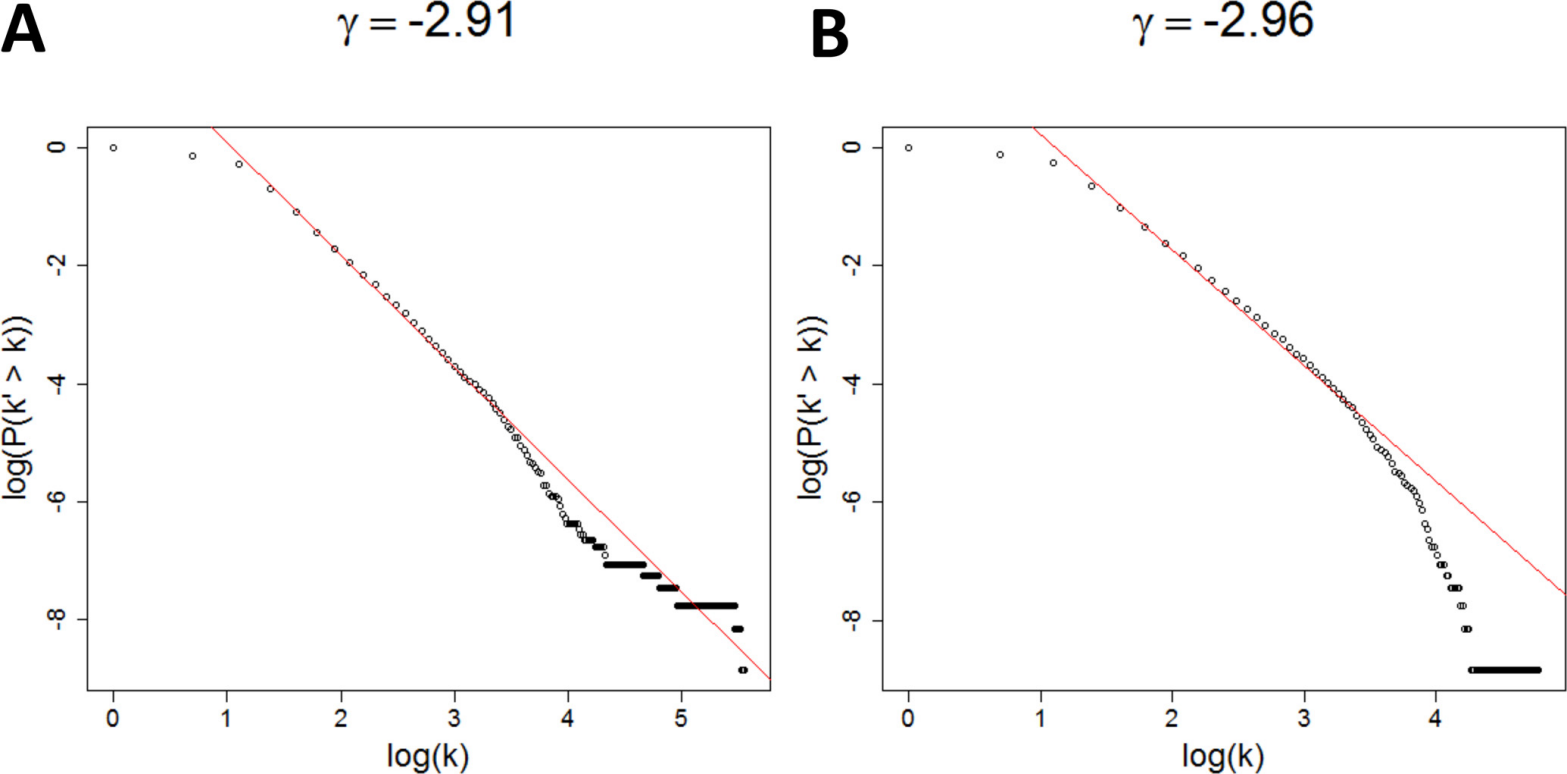
Degree distributions of the BRCA PFN (A) and the LUAD PFN (B). The x-axis is the logarithm of degree k and the y-axis is the logarithm of inverse cumulative degree distribution, P(k’ > k). Red straight line is fitted distribution for P(k^'>k)~k^(γ+1), where γ is the estimated exponent of the underlying degree distribution. Respective γ value is displayed at the top.

As shown in **Fig 5A**, the BRCA PFN is scale-free by following a typical power-law degree distribution with exponent γ ≤ 3, consistent with the frequently observed range of exponents, i.e., 2 ≤ γ ≤ 3, in real-world complex networks [28]. However, the LUAD PFN does not exhibit the characteristics of scale-free degree distribution across all k though the distribution between 3 ≤ k ≤ 50 is scalefree (**Fig 5B**). The decaying tail at k ≥ 50 in the LUAD PFN shows the characteristics of exponential distributions. The diameters of the BRCA and LUAD PFNs are ~11.3, which is in accordance to the hallmark feature of small-world networks with diameters around log(|V|).

In summary, these PFNs possess the hallmark features of complex networks. The qualitative difference between the degree distributions of the two PFNs demonstrate a broad range of network characteristics from scale-free to exponential distributions[23]. These results support the breadth of topological diversity in embedded networks such as PFNs. Several studies of statistical mechanics of embedded networks on topological sphere showed that those networks can possess either exponential degree distribution [31], or scale-free degree distribution with various exponents[23] in thermodynamic limits, depending on the underlying evolutionary dynamics.

### Evaluation of Multicale Clusters

We further compared the clusters derived from MEGENA and those identified by other established clustering and network inference approaches using the TCGA BRCA and LUAD gene expression data (see Data Acquisition and Preprocessing in **S1 Text** for description of BRCA and LUAD data). Specifically, we considered two other types of coexpression networks including weighted co-expression networks (WGCN) and unweighted coexpression networks (FDRN, based on the links at FDR < 0.05)[14, 16] (see **S1 Text** for details) and three established clustering techniques including infomap[38], walktrap[39], and leading eigenvector based spectral clustering[40]. Note that these clustering methods detect coherent clusters in complex network by optimizing for Newman’s modularity Q. Two different similarity measures, MI and PCC, were used for constructing coexpression networks.

Weighted co-expression network analysis (WGCNA) uses its own clustering method which is not suitable for un-weighted networks like PFNs and FDRNs. Towards this end, we compared MEGENA (as a combination of PFN and MCA), WGCNA and the following 6 combinations of networks (PFN and FDRN) and clustering methods (infomap, walktrap, leading eigenvector), PFN + infomap, PFN + walktrap, PFN + leading eigenvector, FDRN + infomap, FDRN + walktrap, and FDRN + leading eigenvector. Furthermore, both MI and PCC were used to construct PFNs, WGCNs and FDRNs.

As there are a few oncogenic signatures available in LUAD, the evaluation of LUAD networks is less comprehensive than that of the BRCA networks. Therefore, we focus on the results from the BRCA data in the main text and report the results from the LUAD data in **S1 Text**.

#### Functional analysis of multiscale clusters

We collected a large number of gene sets associated with known molecular functions and pathways from Molecular Signature DataBase (MSigDB) across GO-BP (Gene Ontology–Biological Processes), GO-CC (Gene Ontology–Cellular Components), GO-MF (Gene Ontology–Molecular Functions), KEGG (Kyoto Encyclopedia of Genes and Genomes) and REACTOME (Reactome database) categories. The significance of the overlap between a given cluster and an annotated gene set was calculated by the Fisher Exact Test (FET), and corrected for multiple testing via Bonferroni correction for the total number of comparisons (i.e. number of clusters Х number of gene sets tested). The detailed results from the enrichment analysis were included in **S1 Data**.

**Fig 6:**
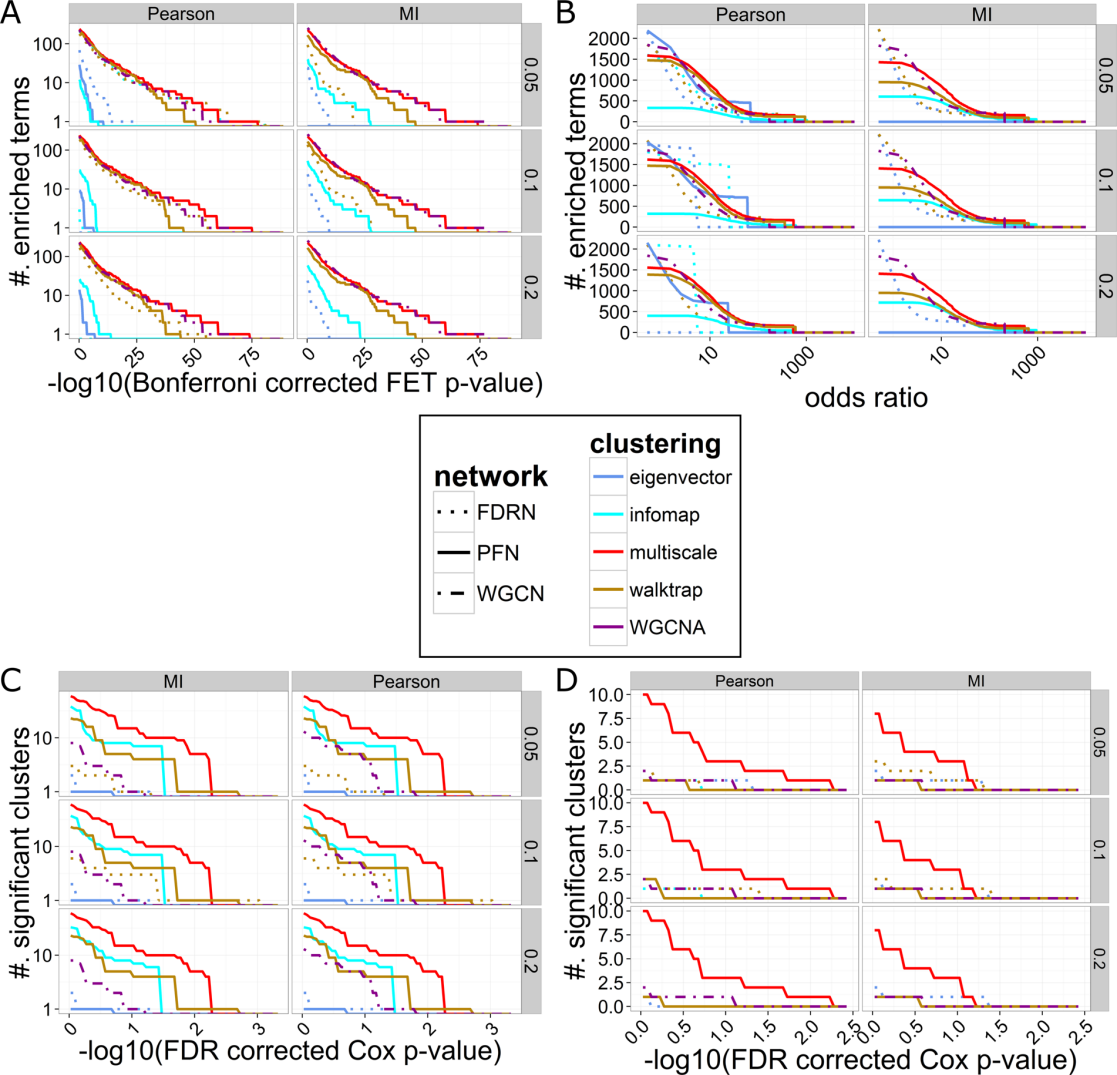
Comparison of MEGENA (as a combination of the multiscale clustering analysis and PFN) and various combinations of the established clustering techniques (eigenvector, infomap, walktrap, WGCNA) and the networks (PFN, FDRN, WGCN) using the TCGA BRCA gene expression data. Two different similarity measures (MI and PCC) were used to perform analyses to compare robustness with respect to difference in measures to evaluate interactions. A) The number of significantly enriched functional/pathway signatures (Bonferroni corrected FET p-values) from MSigDB at various p-value thresholds against. B) Number of significantly enriched functional/pathway signatures from MSigDB at the various odds ratio thresholds. C) Number of clusters predictive of patient survival (based on FDR corrected Cox p-values) at various significance levels. D) Number of clusters predictive of patient survival (based on FDR corrected Cox p-values) and associated to at least one significantly under-represented signatures with Bonferroni corrected FET p-value < 0.05.

As shown in **Fig 6A and 6B**, the multiscale clustering analysis (MCA) has the best performance since the resulting clusters are enriched for the largest number of the annotated gene sets with respect to all significance levels in both MI- and PCC-based networks. More importantly, the MCA-derived clusters show the largest fold enrichment of the BRCA oncogenic signatures. Similar results are observed in PCC-based LUAD networks (see **S3A and S3B Fig**). MCA consistently outperforms the established co-expression network analysis method, WGCNA in both the BRCA and LUAD cases.

Interestingly, the methods that directly optimize for Q (infomap and eigenvector) consistently show better performance on the FDRNs than the PFNs. Q assumes that the random networks follow the configurational model, where edges are shuffled while maintaining the degree sequence [29]. In other words, the underlying random model of Q assumes that the chance of connecting two nodes by an edge is equal for all pairs to *2|E|/(|V|(|V|-1))*, regardless of the degrees of connected nodes. This implies that there is no correlation between the degrees of the nodes sharing an edge in the random network model. However, this assumption does not hold for geometrical networks such as PFNs. We have previously shown that, these random geometrical networks do possess significant degree correlations [23], therefore optimization of Q alone cannot properly address the optimal partition of PFN. On the other hand, FDRN is relatively free from these constraints, and therefore the resulting clusters by optimizing for Q in FDRN reflect the underlying biology better than the case of PFN. On the other hand, the walktrap method resolves this problem by leveraging local random walks instead of Q. Particularly, the walktrap method on the PFNs outperforms the infomap and eigenvector methods on the FDRNs. Altogether, the results imply that the modular structures in PFNs can be better identified by clustering methods that capture local clustering structure, supporting the use of LPI in MEGENA, as described in **Methods**.

#### Prognostic analysis of multiscale clusters

We then examined how each cluster identified from various approaches is associated with the overall survival based on principal component analysis (for details, see Section 4 Cluster-Trait Association Analysis in Methods). As shown in **Fig 6C**, MCA (multiscale) identifies the largest number of significant clusters across a wide range of FDR corrected Cox p-value thresholds, and this is commonly observed in both MI- and PCC-based networks. Although there are relatively more clusters from MCA than other clustering methods due to the hierarchical divisive nature, several clusters from MCA are most predictive of survival. This outstanding performance is also observed in the case of PCC-based LUAD data set (see **S3C Fig**).

We further identified the clusters that were not enriched for any functions/pathway signatures based on Bonferroni corrected FET p-value < 0.05 and the odds ratio > 1. As these clusters have unknown functions, they are termed as unknown-functional clusters (UNC). The UNCs from various approaches are listed in **S2 Data**. We then checked how many of the UNCs identified by each approach were predictive of survival at various thresholds for FDR corrected Cox p-values. As shown in **Fig 6D**, MCA identified the most number of UNCs that were predictive of survival. Such an unambiguous trend was observed in both PCC- and MI-based networks.

In summary, MEGENA as a combination of PFN and MCA identifies the largest number of functional clusters across wide range of cluster sizes in both BRCA and LUAD datasets (see **S4 Fig**). In particular, MEGENA outperforms WGCNA in terms of more significant enrichment for known pathways and better predictive power of survival.

#### Highlight of an adipocytokine-enriched cluster in BRCA

To highlight the findings by MEGENA, we identified 9 functionally annotated clusters (FACs) detected only by MEGENA. These clusters are significantly enriched for some GO-BP/KEGG/REACTOME gene sets that are not enriched in any clusters detected by any other aforementioned approaches based on a threshold of 0.05 for the multiple-test (Bonferroni) corrected p-values. The 9 FACs and their enriched gene sets are shown in **S1 Table**.

**Fig 7:**
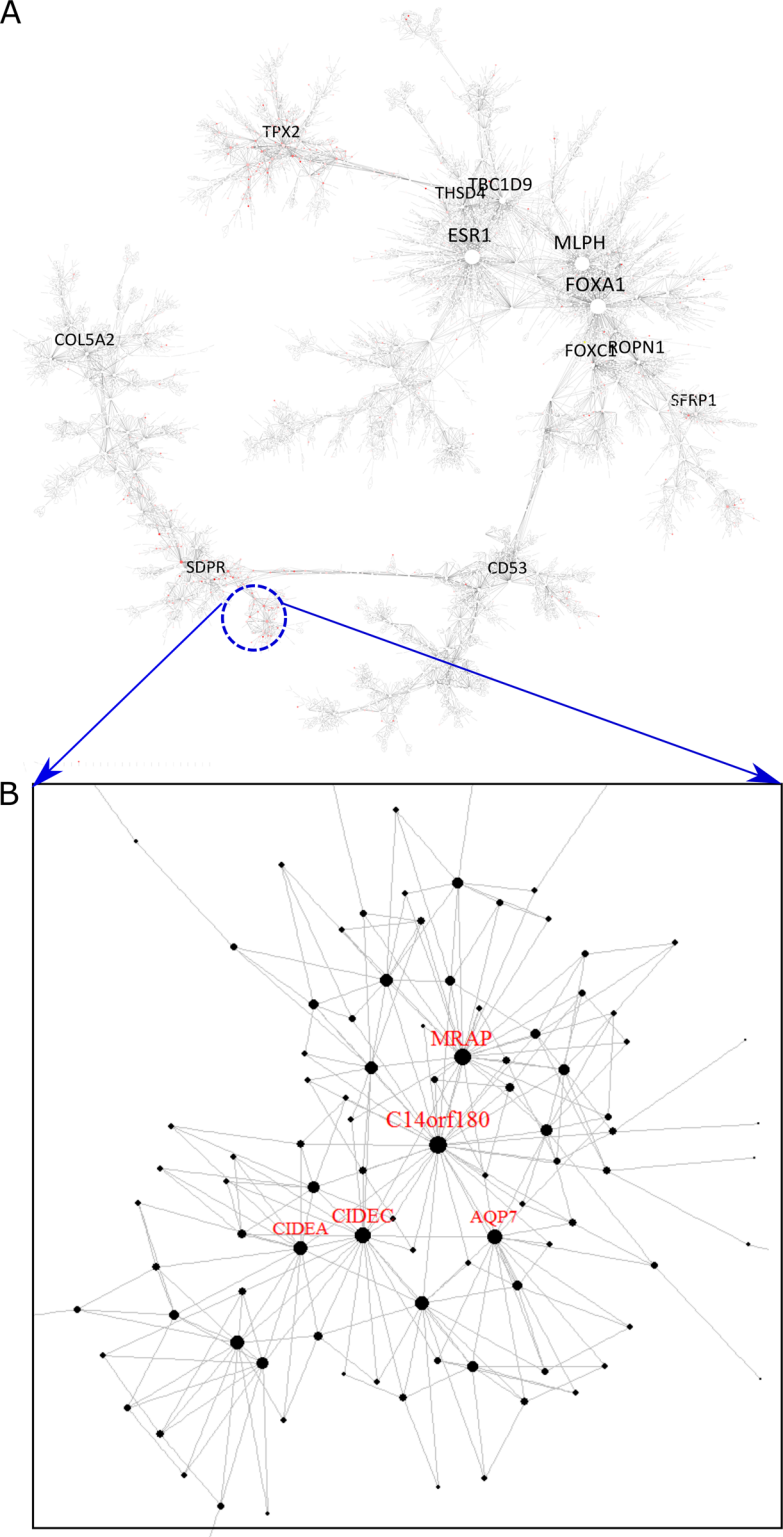
Identification of the adipocytokine-enriched cluster, comp1_56, which was specifically identified by MEGENA. A) The Global BRCA PFN. The nodes in red represent the genes that is predictive of overall survival of LumB patients (Cox p-value <0.05). The blue circle indicates the location of the cluster comp1_56. B) A magnified view of the cluster comp1_56. The nodes with labels are the hubs of the cluster.

**Fig 8:**
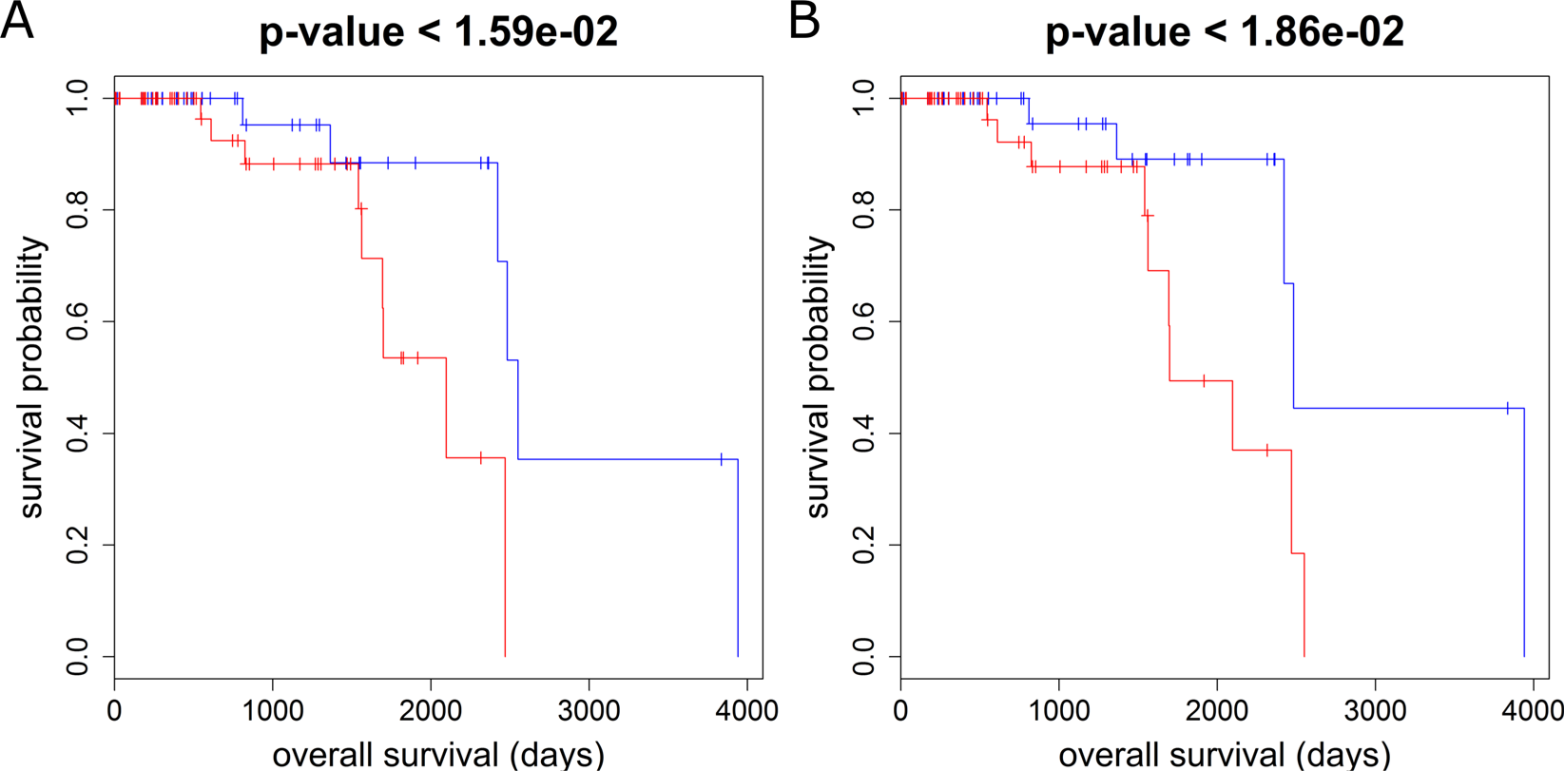
Kaplan-Meier plots of subgroups separated by median expressions of two hub genes *AQP7* (A) and *CIDEC* (B), showing significant logrank p-values. Blue curves showing lower risks correspond to lower expressions, and red curves showing higher risks correspond to higher expressions.

Among these MEGENA-specific FACs, one cluster (comp1_56) is enriched for the genes in adipocytokine signaling pathway (corrected FET p = 0.02, 2.7 fold). The hub genes of this cluster are all significantly associated with the overall survival of the BRCA Luminal-B patients. Luminal-B, a molecular subtype of hormone-receptor positive breast cancers, is associated with higher grade and increased proliferation rate, and has a poorer overall prognosis than its hormone-receptor positive counterpart, Luminal-A [41]**. Fig 7A and 7B** show the localization of genes with univariate Cox p-value < 0.05 for the Luminal B patients’ overall survival at the adipocytokine-enriched cluster. The hub genes of this cluster are all predictive of the Luminal B patients’ overall survival: *AQP7* (Cox p-value < 1.5e-3), *C14orf180* (Cox p-value < 4.4e-3), *CIDEC* (Cox p-value < 1.6e-3), *CIDEA* (Cox p-value < 2.1e-2) and *MRAP* (Cox p-value < 2.2e-2). We further examined the significance of the survival difference between expression median defined subgroups for each hub gene. **Fig 8** shows the Kaplan-Meier plots for the two most predictive hub genes, *AQP7* and *CIDEC*.

These genes were significantly repressed in the tumor samples in comparison to the matched normal samples for Luminal-B patients: *CIDEC* (fold change (FC) = 2.5E-2, T-test p-value (p) = 3.5E-4), *CIDEA* (FC = 4.9E-2, p = 1.4E-3), *AQP7* (FC = 4.7E-2, p = 7.1E-4), *MRAP* (FC = 4.3E-2, p = 1.2E-3) and *C14orf180* (FC = 4.7E-2, p = 5E-4). This is in agreement with the previous findings that *AQP7* expression was down-regulated in breast tumor[42] and *CIDEA* and *CIDEC* are cell death-inducing DFFA-like effectors to activate apoptosis[43]. Particularly, *CIDEA* and *CIDEC* are involved in adipose tissue loss in cancer cachexia[44], an important, negative prognostic marker that has been linked to systemic inflammation and cell death[45].

In summary, these findings suggest that adipocytokine signaling pathways may play an important role in Luminal B subtype of breast cancer though the exact mechanisms need further experimental validation. Interestingly, the expression of the hub genes in the adipocytokine cluster/subnetwork were not significantly associated with the overall survival outcome of the Luminal A patients. Therefore, MEGENA is capable of capturing finer-scale functional subnetworks to stratify a breast cancer subtype into the subgroups with prognostic significance.

### Multiscale Organizations in PFNs

In this section, we will explore the multiscale clustering structures in PFNs constructed by MEGENA. Here, we mainly focus on the PCC-based BRCA PFN as it showed slightly better performance than the MI-based network (**Fig 3B–3E**).

#### Multiscale organization of functions/pathways

**Fig 9:**
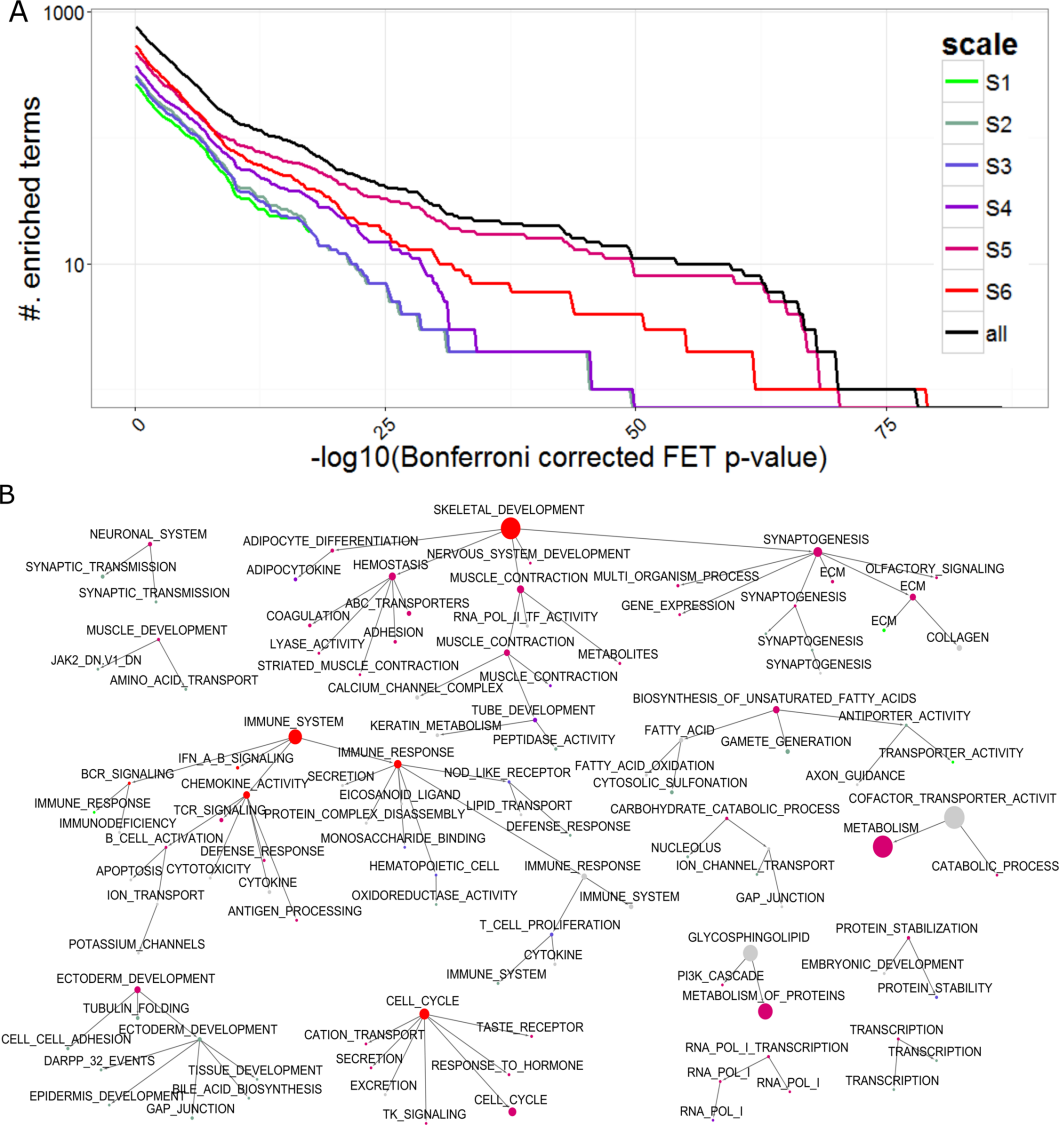
Hierarchical organization of functions and signaling pathways corresponding to the multiscale clusters identified by MEGENA. A) Comparison of number of significantly enriched functions and pathway signatures across clusters identified at different scale groups. The scale groups identified from MHA are colored according to the legend, and “all” denotes collection of clusters across the scale groups. B) Multiscale organization of clusters in PFN. Each node is a cluster identified by multiscale clustering in PFN, where the node size is proportional to the cluster size, node color coincides with the cluster group color scheme in A, and node labels indicate most enriched function/signaling pathway for individual clusters. A directed link a→b indicates b is a sub-cluster of a.

We performed MHA on the BRCA PFN to identify the groups of scales that had similar interaction patterns and shared highly connected hubs across different scales. Six distinctive scale groups were identified: S1 (0.03 ≤ α ≤ 0.48), S2 (0.5 ≤ α ≤ 0.82), S3 (0.87 ≤ α ≤ 1), S4 (1.01 ≤ α ≤ 1.29), S5 (1.3 ≤ α ≤ 1.82) and S6 (1.83 ≤ α ≤ 6.8). Biological relevance of each scale group was evaluated by the number of significantly enriched MSigDB gene sets. We compared the performance of the clusters at each scale group and that of the clusters across all scale groups. **Fig 9A** shows that the combination of all the clusters across the different scale groups consistently outperforms the individual scale groups across almost the entire range of significance levels. Interestingly, the clusters at the scale S5 (1.30 ≤ α ≤ 1.82) show the best performance when compared against other scale groups. The clusters identified at the finest scale of S5 (α = 1.3) are shown in **Fig 4**.

To further understand the biology of the clusters at the different scales, we annotate each cluster with its most enriched function or pathway and then build up a cluster hierarchy based on the parent-child relationships (e.g., a child cluster is a subset of the parent cluster). As shown in **Fig 9B**, the cluster hierarchy clearly displays biological relevance of the multiscale/multisresolution organization patterns in the BRCA PFN, by showing hierarchical aggregation of specific functions and pathways to more general terms. For instance, the cluster annotated as SKELETAL_DEVELOPMENT (FET p-value<2.7e-08, 2.48 fold enrichment) is the parent of the clusters with more specific functional categories associated with development/differentiation: HEMOSTASIS (P<2.49e-05, 5.35 fold), ADIPOCYTE_DIFFERENTIATION (P<3.95e-08, 19.42 fold), SYNAPTOGENESIS (P<4.32e-08, 7.18 fold), and MUSCLE_CONTRACTION (P<1.503e-08, 15.22 fold).

In the case of the PCC-based LUAD PFN, there are twelve distinct scale groups (0.2 ≤ α ≤ 3.2) (see **S5A and S5B Fig**). Again we observed the superior performance of the clusters across all scale groups over any individual scale (see **S5C Fig**).

#### Identification of novel key drivers via MHA

Having validated that some known interactions are present in the BRCA PFN via the oncogenic signatures, we hypothesize that hub genes of the BRCA PFN are more likely to be key drivers of BRCA etiology, and further explore biological significance of novel key drivers of the network.

**Fig 10:**
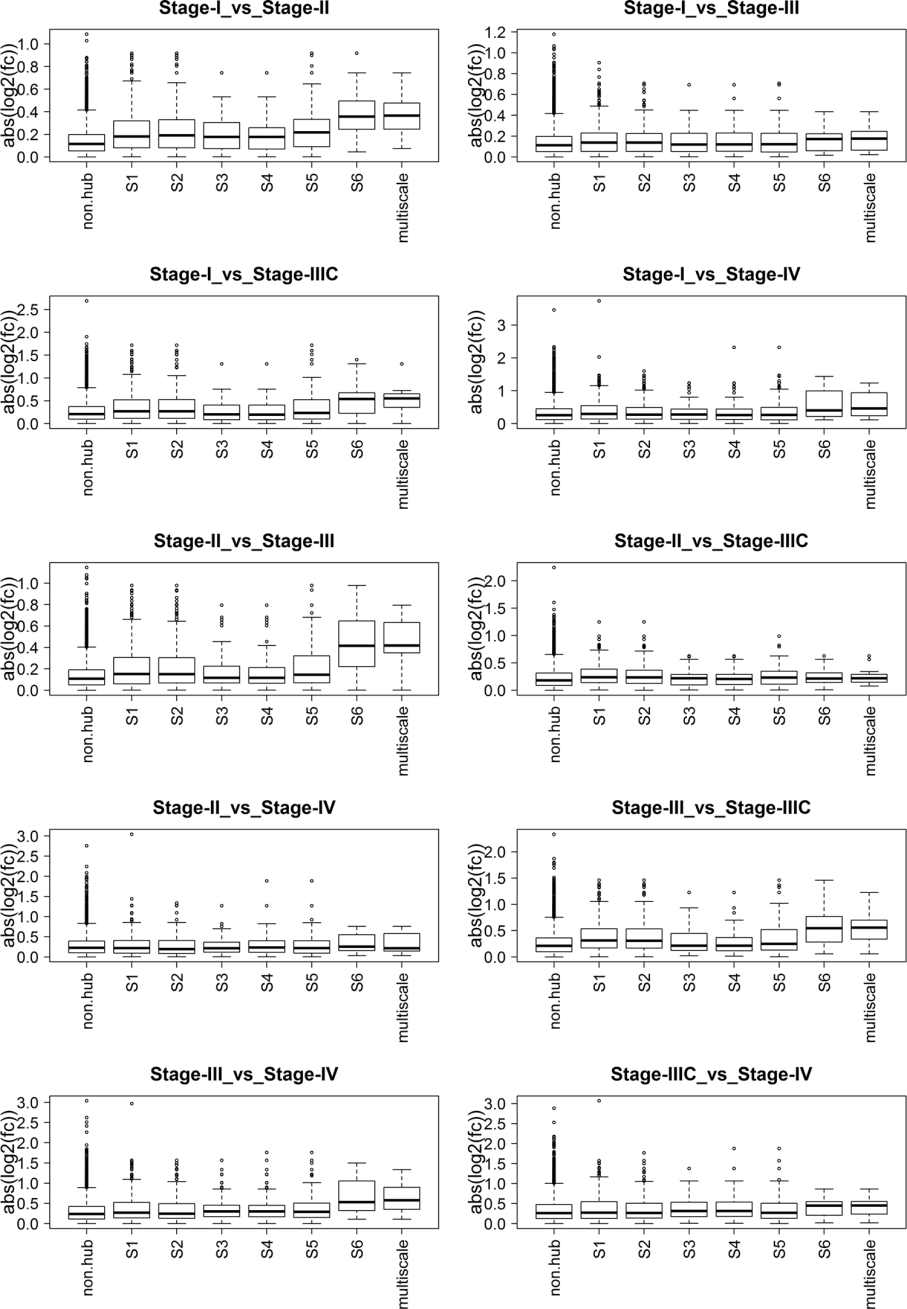
Comparison of expression fold changes (FC) of the hub genes and non-hub genes between different cancer stages in BRCA, against lists of genes identified by mutiscale hub analysis, where fc denotes expression fold change. The numeric labels on x-axis represent the ranges of α values defining the resolution levels of the hubs, “multiscale” represents intersection of hub genes across different scales, and “non.hub” represents the rest of genes.

To verify biological significance of hub genes detected by MHA, we compared expression fold changes of network hubs between different cancer stages, to those of the non-hub genes. We first identified the hub genes at each scale, and then intersected the hub gene sets at the different scales to identify a more stringent hub set, denoted as “multiscale hubs”. We then evaluated the significance of the difference between the fold change distributions from each hub set and the corresponding non-hub genes using the Kolmogorov-Smirnov (KS) test. **Fig 10** compares the distributions of expression fold changes in the two groups with respect to different stages of breast cancer. **S6 Fig** shows p-values from the KS test.

In many cases, the hubs show significantly higher expression fold changes than the non-hub genes, and the average fold changes are greater for hubs at larger scales. Particularly, the multiscale hubs show the highest average fold changes, suggesting that they may be transcriptomic drivers of breast cancer progression. There are 14 multiscale hub genes including *ROPN1*, *TPX2*, *TEKT1*, *FOXA1*, *ESR1*, *CCNT1*, *SDPR*, *THSD4*, *MLPH*, *TBC1D9*, *FOXC1*, *SPDEF*, *SFRP1*, and *AR*. Many of these genes are known to play important roles in breast cancer etiology. For instance, *AR* and *ESR1* are well-established endocrine receptors in breast cancer [46, 47]. *FOXA1* is a pioneer transcription cofactor for *ESR1*, opening chromatin containing *ESR1* target genes[48]. In ER- breast cancers, *FOXA1* may promote androgen signaling and may contribute to the development of resistance to anti-androgen therapy [46]. *SPDEF*, a target gene of *ESR1*, overexpressed in breast and other solid tumors, was shown to be associated with worse outcomes in patients with ER+ breast cancers and to be critical for the survival of ER+ breast cancer cells *in vitro* [49]. *SPDEF* was upregulated in ER^+^ breast cancer cell models of estrogen-deprivation resistance and tamoxifen resistance[50]. *TPX2* is an established regulator in spindle assembly and DNA damage response across many solid tumors[51], and indeed is a key regulator of the cell-cycle cluster identified by MEGENA. *FOXC1*, a prognostic biomarker of basal-like breast cancer patients[52] is a key regulator of *NF*-*kappaB* signaling pathways in basal-like breast cancer cells[53] and promotes breast cancer invasion[54]. *SFRP1* is a known inhibitor of *Wnt* pathway and tumor suppressor gene, which is epigenetically silenced in a variety of tumors including breast cancer[55].

**Fig 11:**
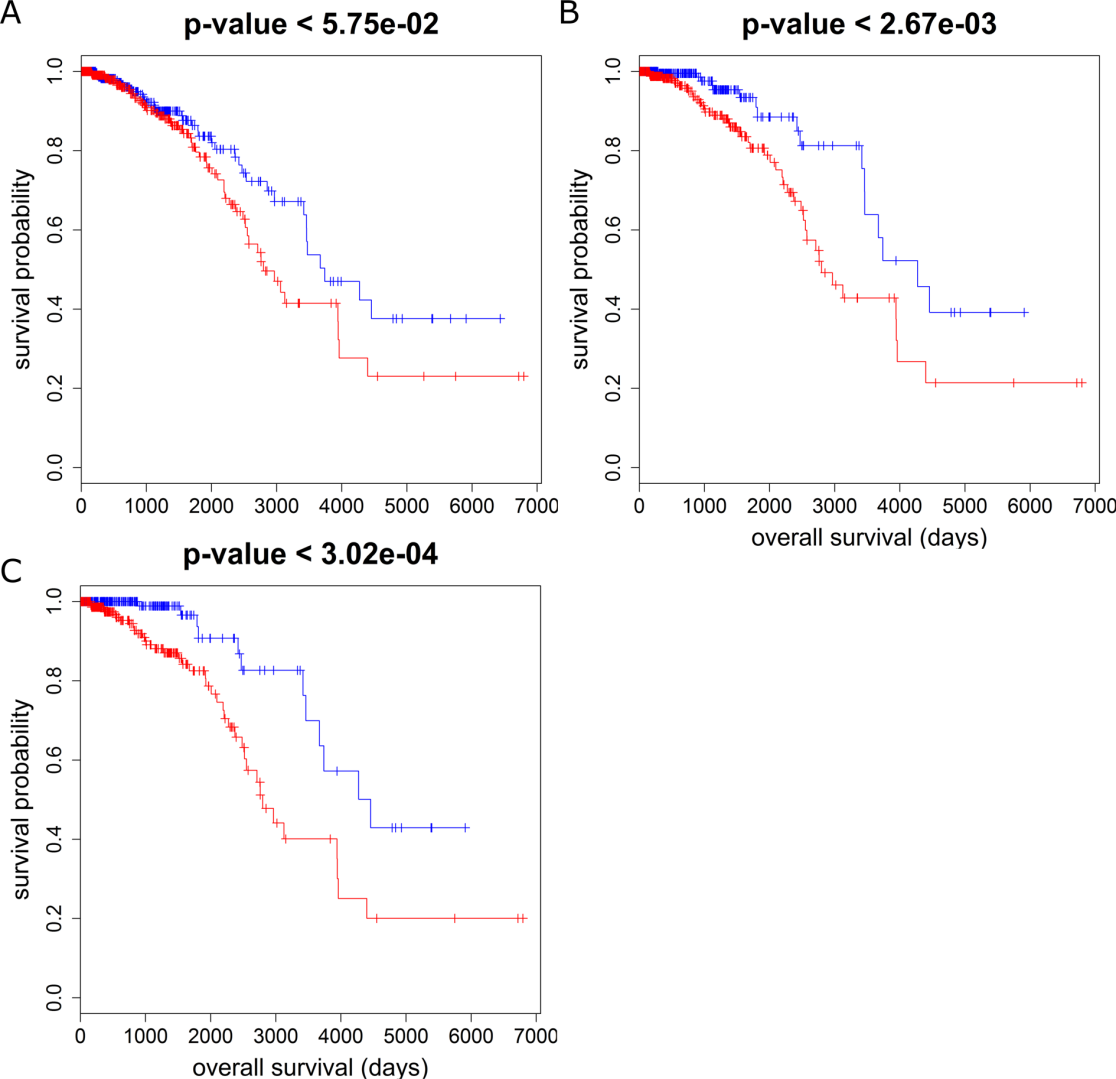
Kaplan-Meier plots of the subgroups defined by median expression of *ROPN1* in A) all the patients, B) the ER+ patients and C) the PR+ patients. Blue and red curves correspond to the lower and higher expression levels of *ROPN1*, respectively.

This multiscale hub set also includes a number of novel genes as promising targets of breast cancer for further studies. One example is *ROPN1*, which is an immediate neighborhood of *FOXC1* and *FOXA1*. *ROPN1*, also known as *ropporin*, is a cancer-testis antigen[56] and a potential immune-therapeutic target for multiple myeloma as Chiriva-Internati *et al*. generated human leukocyte antigen class I-restricted cytotoxic lymphocytes to kill autologous multiple myeloma cells[57]. *ROPN1* is significantly associated with the overall survival for all the BRCA patients in TCGA (Cox p-value < 2.6e-2, logrank p-value < 5.8 e-2), PR+ (Cox p-value < 9.7e-5, logrank p-value < 3.0e-4), and ER+ (Cox p-value < 3.3e-4, logrank p-value < 2.7e-4). Higher expression of *ROPN1* was associated to better prognosis. Furthermore, *ROPN1* is mostly down-regulated in tumor samples in comparison to normal samples (FDR corrected test p-value < 8.4e-14, the ratio of the average expression in the tumor samples to that in the adjacent normal samples = 0.14). Fig 11 shows a significant survival difference between the *ROPN1*-low and -high groups for all, ER+ and PR+ patients. Moreover, *ROPN1* is predictive of survival of the Her2 subtype identified by PAM50 biomarkers (Cox p-value < 2.2e-3) [58]. These results suggest *ROPN1* as a desirable immunotherapeutic target for further validation.

We also performed MHA on the PFN clusters based in the TCGA LUAD data set to verify the findings in the BRCA data set shown in **S7** and **S8 Figs**. We again observed a similar pattern to the BRCA PFN, i.e., the multiscale hubs of the LUAD PFN have significant expression fold changes. *TPX2*, *C16orf89* and *GJB3* emerged as the multiscale hubs present at all scale groups in the LUAD PFN. Notably, *GPR116* and *HOPX* directly connect with *C16orf89* in the LUAD PFN and are hubs at several scales. In particular, *HOPX* (HOP homeobox) is a lineage-specific transcriptional regulator of differentiation and has been shown to control the fate of LUAD progression where the cooperative expression of *HOPX* with *GATA6* limits metastatic competence of LUAD cells[59]. *GPR116*, an essential regulator of lung surfactant homeostasis, was shown to play a crucial role in preventing alveolar collapse through its ability to reduce surface tension[60, 61]. We further showed that the DEG signatures from *HOPX* and *GATA6* double-knockdown in the lung adenocarcinoma cell lines[59] and *GPR116* knockdown in the murine type II alveolar epithelial cells from the *Gpr116* knockout mice (GSE41417)[62] were significantly enriched in *HOPX* and *Gpr116’s* neighborhoods, respectively, (see **S9 Fig**). Altogether, these results suggest *C16orf89* as a novel therapeutic target in preventing lineage-specific metastasis in LUAD for murine type II alveolar epithelial tissue.

## Discussion

We developed a novel framework, Multiscale Embedded Gene Co-expression Network Analysis (MEGENA), to infer gene co-expression networks, by implementing a parallelized algorithm for embedding co-expression networks on topological sphere and a new clustering analysis algorithm to detect coherent clusters at various compactness scales. MEGENA constructs a co-expression network by enforcing an objective criterion of “embeddability” of candidate connections on topological sphere, and thus reduces the inherent redundancy of pair-wise interactions. MEGENA-derived networks possess the hallmarks of complex networks [21, 23, 31]. Application of MEGENA and the state-of-the-art network inference approaches to the simulated data with gold standard networks shows that the MEGENA-derived PFNs have the best and most stable performance. The outstanding performance of MEGENA was further demonstrated in the significant overlap between the siRNA knock-down signatures of a large number of functionally important TFs and their inferred network signatures.

We showed that the novel multiscale clustering technique in MEGENA can identify biologically more meaningful and relevant coexpressed gene clusters than established network clustering methods such as infomap, walktrap, leading eigenvector spectral clustering, and WGCNA.

We highlighted the novel insights from the multiscale approach to decipher gene-gene interaction networks. Key drivers/hubs of MEGENA-derived networks were further identified by MHA, a key procedure in MEGENA. We identified *ROPN1* and *C16orf89* as the novel candidate drivers for BRCA and LUAD, respectively.

As an alternative approach to analyzing big Omics data, MEGENA has demonstrated competitive performances and will have a great potential in unraveling novel pathways and key regulators in complex diseases. There are rooms to further improve MEGENA. Currently, we are extending the network construction algorithm to higher genus to account for more complex interaction patterns as hyperbolic surfaces with higher genus are able to accommodate for more complexity [31].

### Software Availability

The *MEGENA* R package for Windows can be downloaded from here: http://research.mssm.edu/multiscalenetwork/packages/MEGENA_1.1.zip

The *MEGENA* R package for Linux can be downloaded from here: http://research.mssm.edu/multiscalenetwork/packages/MEGENA_1.1.tar.gz

## Methods

### Fast Planar Filtered Network Construction

A key component of MEGENA is the construction of Planar Filtered Networks (PFNs). Here we developed a new procedure named FPFNC to substantially improve the existing PMFG in terms of efficiency and scalability. Specifically, we first introduced a parallelization process for testing planarity, and then implemented early termination options to construct ‘nearly maximal’ embedded networks which prevent inclusion of less informative but computationally expensive links. The procedure for constructing a PFN is detailed below.

#### Compute similarities between gene expression profiles

Given a gene expression dataset with N genes and M samples, we first compute the similarity between any two genes. A number of similarity measurements such as correlation, Euclidean distance and mutual information can be employed to compute the similarity between expression profiles. MEGENA can take as input similarities from any similarity measurement. Comparison of various similarity measurements is beyond the scope of this paper. Gene-gene similarities are then filtered by False Discovery Rate (FDR) to minimize the impact of false positives. FDR is computed by permuting gene expression matrix across the samples (global FDR), or by directly calculating pairwise nominal p-values by Fisher’s Z-transformation. In the current implementation of FPFNC, we set FDR < 0.05 as the default threshold for filtering similarities.

#### Construct PFN

**Fig 12:**
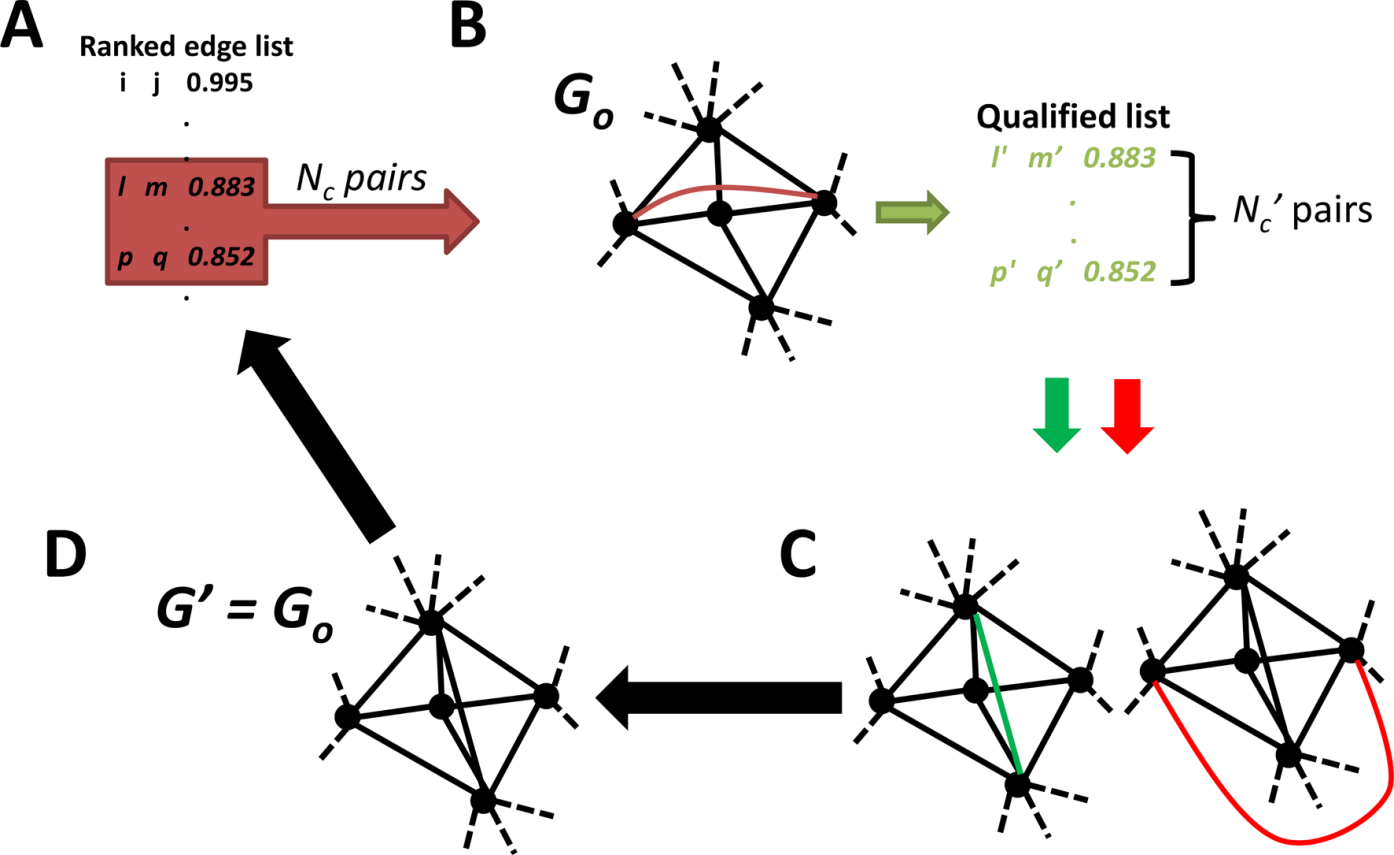
Fast PFN construction. A parallelized screening procedure is developed to extract a subset of gene pairs which are highly likely to be embedded. A) FPFNC begins with a rank-ordered list of association pairs. B) Then a subset of *N*_*c*_ pairs undergo parallelized quality control by their embeddability on a single platform of *G*_*o*_ to identify the pairs which are more likely embedded in the subsequent network construction steps. C) These screened set of *N* ^*’*^_*c*_ pairs are then tested on the growing embedded network subsequently. D) A final updated network *G* ^*’*^, which will be used as *G*_*o*_ on the next cycle. The whole processes are repeated until the defined criterion for termination is met.

The existing PMFG algorithm embeds an input network onto a topological sphere by the following steps [21] (**Fig 12)**:

It begins from the empty network *G*_*o*_(*V*_*o*_,*E*_*o*_) where nodes are completely disconnected, i.e. *E*_*o*_ = ∅.
Then, it rank-orders gene pairs by their co-expression similarities, and iteratively test planarity of each pair *ij* by Boyer-Myrvold algorithm.
If a pair *ij* passes the planarity test, this results to update *G*_*o*_ by adding the pair as a link in the network, i.e. *E*_*f*_ = *E*_*o*_ ∪ {*ij*}. This is equivalent to embedding *ij* onto the sphere.
i) ~ iii) is repeated in a serial manner until the maximal number of edges *|E*_*max*_ *| = 3(|V*_*o*_ *|-2)* is reached, and results in *G*_*f*_ *(V*_*f*_, *E*_*f*_ *)* where *V*_*o*_ *= V*_*f*_ and *|E*_*f*_ *| = |E*_*max*_ *|*.

The resulting network *G*_*f*_ is an embedded network on a topological sphere where every edge can be drawn without crossing another.

To speed up the planarity test, we designed a parallelized screening procedure (PCP) to extract a subset of gene pairs that are more likely to be embedded. The underlying premise of PCP is explained as follows. If *G*′(*V*′,*E*′) with *E*′ = *E*_*o*_ ∪ {*ij*} is embeddable, then *G*″(*V*′,*E*″) with (*E*″ − {*ij*}) ⊆ *E*_*o*_ is embeddable. Intuitively speaking, we are utilizing the fact that any subnetwork of a planar network is always planar. Therefore, if *ij* is one of edges included in the final network *G*_*f*_, the combination of any subnetwork in *G*_*f*_ or *G*_*o*_ with the edge *ij* must be embeddable since the combination is also a subnetwork of a planar network.

This allows to use *G*_*o*_ as a platform to test the quality of *N*_*c*_ individual pairs prior to the link embedding step (the step (iii) in PMFG) in PCP where each of *N*_*c*_ pairs are combined with *G*_*o*_ and tested for planarity in parallel. The qualified pairs after PCP proceed to the serial embedding step in PMFG, finally yielding to the updated network *G*_*f*_. This process is also shown in **Fig 12**.

Currently, we implemented *N*_*c*_ *= 1000 x N*_*core*_ where *N*_*core*_ is the number of cores used in parallel computation. Furthermore, we have set PCP to start when the acceptance rate of each edge into *G*_*f*_ falls below 10%, where acceptance rate is defined as the number of accepted pairs by the number of tested pairs. We recommend that users follow the current parameters as we show that PCP with current parameters improves the overall computation time effectively as discussed in Section 1 in Results.

#### Terminate PFN construction

Early termination conditions are set up to further bypass unnecessary computations to embed less informative pairs that are not filtered out by Step 1.1 while keeping sufficient information for network construction. By setting up reasonable termination options, the resulting PFN still harbors sufficient information. The construction process will be terminated if one of the following conditions is satisfied: (i) a network is maximally embedded (identical to PMFG when Step 1.1 is bypassed)[21]; (ii) an embedded network reaches a certain saturation where the mathematically permitted maximum number of links is *3(|V|-2)*; (iii) the number of rejected pairs per one embedded link reaches a certain threshold. For the condition (iii), we set the threshold for the number of rejected links as *20|V|*, corresponding to FDR < 0.05. Empirically, we found out that the condition (ii) with 90~95% of the maximum number of links or the condition (iii) with the default number of rejected links can provide sufficient results with minimal information loss.

### Multiscale Clustering Analysis (MCA) on PFN

As most biological networks exhibit highly modular and yet hierarchical organizations[3, 7], we next set about to identify coherent modular structures in PFNs. It has been well-known that characterization of the organization patterns in complex networks cannot be done by a single perspective, but requires a combination of multiple distinctive and diverse features[8].

#### Measures of compactness and local clusteredness

We perform multiscale clustering analysis of PFNs by optimizing a number of key network features including within-cluster compactness, local clustering structures, overall modularity, and other network-theoretic measures.

Specifically, within-cluster compactness is measured via the shortest path distances (SPD) on PFN[28], local clustering structure via Local Path Index (LPI)[63], and overall modularity via Q[29]. SPD is defined as distances of shortest geodesic paths among all nodes in a given network[64], thus can be naturally used to represent the degree of compactness for a given set of nodes by summarizing overall distances between the nodes on the network. However, SPD tends to over-emphasize hierarchical organization of nodes, and thus ignores clustering structures[65].

In order to mitigate this problem, we chose LPI to incorporate local clustering structures in PFN. LPI is defined as A^2^ + ϵA^3^, where A is the binary adjacency matrix of the network, ϵ is a free parameter, and [A^n^]_ij_ is the number of paths of n steps linking nodes i and j [63]. LPI is a quasi-local measure that evaluates structural similarity between two nodes on a network by accounting for all paths of lengths 2 and 3 between any two nodes. LPI with ϵ = 0.01 in the context of link prediction has been shown to perform superior to some of already established network theoretic similarity measures in many real-world cases[63, 66]. Particularly, the abundance of 3-cliques and 4-cliques in PFN works synergistically with LPI for the following reasons: a) a majority of the 2- and 3-step paths are confined within these cliques, and b) organization patterns of adjacent 3-cliques and 4-cliques provide rich clustering information of PFN[22, 23, 27], and c) the adjacency information can be readily captured by LPI by paths that span between different cliques. Furthermore, the computation time for LPI is linear with the size of network[63], making it suitable for large-scale co-expression network analysis. Lastly, we leveraged the capability of Q to evaluate significance of within-cluster connectivity in tandem with between-cluster connectivity to guide proper splitting of networks into sub-clusters.

We introduced a network compactness measure ν(α) as a function of a resolution parameter α, to capture the cohesiveness of modular structures in PFN at different resolutions (or scales) defined by α. Particularly, we leveraged geometrical characteristics of PFN that retains similarity between two nodes by means of geodesic path distance such that coherent clusters exist as connected subgraphs with higher local clustering coefficients[21]. Furthermore, these geometrical networks exhibit a broad spectrum of compactness characteristics from loose-world to semi-ultra small-world[23], and thus they allow different degrees of compactness to co-exist. **Fig 1B** presents an overview of the procedure in MCA and the details of MCA are described in the following subsections.

#### Network split: k-split

At each iteration, a nested split is performed on each cluster to derive an optimal split with respect to the initial cluster via k-medoids clustering which detects k optimal clusters by minimizing the SPD based intra-cluster distance[67]. However, SPD based evaluation of the partitions at each k does not take into account clusteredness of local topology, leading to misclassification of the nodes at the boundaries between adjacent clusters. To remedy this problem, we designed a procedure to update the boundaries of the clusters derived from a partition at each k. Boundary nodes are those with immediate neighbors from at least two different clusters. Let *V* ^*bnd*^ be a set of boundary nodes. Then, for a node *i ϵ V* ^*bnd*^, its cluster membership to *V*_*l*_ is defined via the following equation,

$$\mu_{V_{l}}^{i}=\max_{V_{l^{\prime }}}\left\{\mu_{V_{l^{\prime }}}^{i}|\mu_{V_{l^{\prime }}}^{i}=\sum_{j\in V_{l^{\prime }}}LPI_{ij}\right\}$$ (2)

where membership of *i* is assigned to *V*_*l*_.

**Fig 13:**
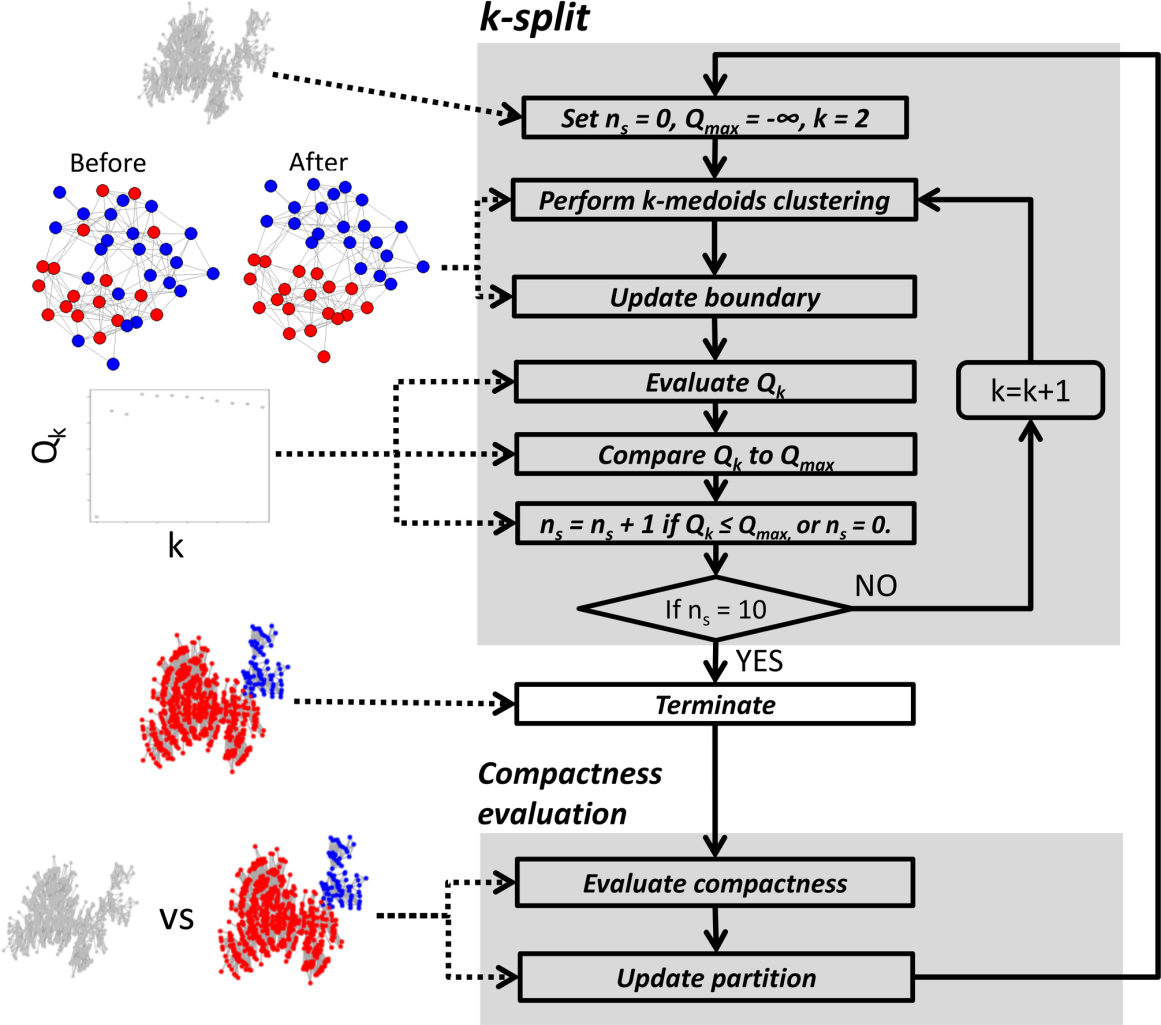
Flow chart of the clustering analysis procedure for each value of compactness resolution parameter, α. The upper panel illustrates the k-split procedure within each cluster to detect optimal sub-clusters. The lower panel describes the compactness evaluation procedure (CEP) after k-split. CEP compares the parent cluster prior to k-split with the sub-clusters after k-split by means of the compactness measure, *ν*_*l*_, and updates the partition accordingly. On the left, each step is illustrated by a graphical toy example. From the top, the pictures correspond to: the initial network subject to clustering, correct classification of boundary nodes by BDP (Before: before BDP, After: correction after BDP), identification of the optimal k via modularity Q_k_, final clusters, and comparison between initial network and sub-clusters via compactness. These steps are iterated for all clusters from the newly updated partition until no further update can be made.

The Eq (1) assigns a given node *i* to a new cluster in which the node *i* has the maximal number of interactions as determined by LPI. This boundary detection procedure (BDP) is iterated until there is no change in cluster membership, therefore leading to a stable partition. This process is illustrated in **Fig 13** in the step “update boundary”, showing a toy example where misclassified red nodes in “Before” panel are correctly assigned in the “After” panel after BDP.

As network split requires determination of the optimal k value, we utilized an established measure of clusteredness on networks, Newman’s modularity, Q[29]. Specifically, we repeat the k-medoids partition and BDP for a range of k values until no further optimal solution by Q is available in the interval [k’—dk, k’], where dk is set as 10. In other words, we search for the optimal partition determined by Q within 10 further partitions, and repeat the search until no better solutions are detected. Although we do not have the data for systematic evaluation on setting the appropriate dk value, we did not observe much differences in resulting clusters in the exemplified BRCA and LUAD PFNs for dk ≥ 10, and dk = 10 often succeeds to explore up to k ≈ 60 in these cases. Given that the clustering is a hierarchical divisive method and 2 ≤ dk ≤ 6 are often the number of clusters explored for each split in hierarchical divisive methods [15, 40], we recommend dk = 10 is sufficient to effectively identify these clusters.

#### Identification of significantly compact sub-clusters

Upon splitting a network *G*_*o*_(*V*_*o*_,*E*_*o*_) into clusters, each cluster is evaluated by the compactness measure defined as the average shortest path distance within a cluster *V*_*l*_, normalized by the logarithm of the cluster size,

$$\upsilon_{l}=\frac{\sum_{i,j\in V_{l}}SPD_{ij}}{\log{\left(\left|V_{l}\right|\right)}^{\alpha }\left(\left|V_{l}\right|\left(\left|V_{l}\right|-1\right)/2\right)}=\frac{\bar{SPD}}{\log{\left(\left|V_{l}\right|\right)}^{\alpha }},$$ (3)

where α is a scaling parameter to control the degree of compactness. Normalization by log(|V|)^α^ is in accordance to the scaling effect from the diameter, $\hat{SPD}$, of geometrical networks such as PFNs. In the thermodynamic limit of |V| → ∞, geometrical networks can be modeled by means of a stochastic network model where small clusters of “bubbles” aggregate to give characteristic features of real-world complex networks, i.e., $\hat{SPD}∼{\log\left(|V|\right)}^{\alpha }$, where α ≥ 1, a hallmark feature of small-world property[23]. However, $\hat{SPD}$ does not reflect network structural information since it relies on the minimum of the longest pair-wise shortest path lengths. To mitigate this, we utilize $\bar{SPD}$ to incorporate all information between all pairs of nodes, and take the scaling effect from $\hat{SPD}$ as an upper bound of network compactness.

For a given network subject to split, a split is obtained by maximizing for Q that is independent of α. Then, at a given α, the following criterion determines the acceptance or rejection of the split:

$$\{\begin{array}{c} \text{Accept}:\text{if}\;\nu_{\text{o}}>\upsilon_{\text{l}}\;\text{for}\;\text{any}\;\text{l},\\ \text{Reject}:\text{if}\;\nu_{\text{o}}<\upsilon_{\text{l}}\;\text{for}\;\text{all}\;\text{l}, \end{array}$$ (4)

where, *ν*_*o*_ is the compactness of the parent cluster. The criterion implies that, for a given *α*, a split that results in at least one cluster *l* with improved *ν*_*l*_ in comparison with *G*_*o*_ is considered as valid.

In order to efficiently search for valid splits across all α, we leveraged the independence between the split detection via Q, and the split acceptance criterion. For a given network *G*_*o*_, we define a characteristic *α* value as the one that can identify a more compact cluster *l* than *G*_*o*_:

$$\alpha_{l}^{c}≔\underset{}{\max}\left\{\alpha_{l}|\nu_{o}>\frac{\bar{SPD}}{\log{\left(\left|V_{l}\right|\right)}^{\alpha_{l}}}\;\text{with}\;\alpha_{o}^{c}\geq \alpha_{l}\right\}$$ (5)

Calculation of $\alpha_{l}^{c}$ for all the clusters from a split, allows us to identify α values at which the split is accepted according to the criterion (4).

Individual sub-clusters from an accepted split are evaluated by calculating the statistical significance of *ν*_*l*_ at a scale $\alpha =\alpha_{l}^{c}$. Specifically, a random PFN is first generated by shuffling the link weights of the parent cluster. Then, *|V*_*l*_ *|* nodes are sampled for 100 times from the random parent, and the compactness values for these random networks, $\nu_{l}^{\prime }$, are calculated to estimate significance p-value of *ν*_*l*_. Random node insertion moves (namely T2 moves) are utilized to generate random PFNs which exhibit scale-free degree distributions and small-worldness in thermodynamic limits [23].

#### Termination of algorithm

The search for sub-clusters is performed iteratively until no valid sub-clusters can be further identified under the following conditions:

- no sub-clusters can be further identified as more compact than respective parent clusters at any α, or
- no further sub-clusters show significant compactness (P>0.05).

### Multiscale Hub Analysis (MHA)

We developed a Multiscale Hub Analysis (MHA) procedure to identify highly connected nodes at each scale defined by α and across all the scales. MHA identifies the nodes with significantly high connectivity within each significant clusters previously identified through the following steps: 1) *Group the scales* that show similar within-cluster connectivity patterns, 2) *Identify hubs at each scale*, and 3) *identify multiscale hubs* by combining significance scores of individual nodes across all different scales. The procedure is detailed in the following subsection.

#### Grouping similar scales

At each scale α, we define within-cluster connectivity of node *v*_*i*_ as

$${\text{c}}^{\text{w}}\left(v_{i},\alpha \right)=\sum_{v_{j}\in V_{l}^{\alpha }}A\left(v_{i},v_{j}\right)$$ (6)

where $V_{l}^{\alpha }$ is the set of nodes in the cluster *l* at the scale *α*, and *A*(*ν*_*i*_,*ν*_*j*_) = *w* is the adjacency matrix denoting the weight of link connecting *v*_*i*_ and *v*_*j*_. Combining c^*w*^(*ν*_*i*_, *α*) across all nodes and α values, we obtain C^*w*^(*V*,A) where V is the set of all nodes in PFN, and Φ is a set of α values that have at least one significant split.

Then, k-medoids clustering is performed for each *α* ∈ Φ. The optimal number of clusters, k, is then estimated by summarizing results from multiple established internal validity indices since a single validity index may emphasize only a specific criterion and may not provide good quality clusters [68]. Specifically, we utilized a number of internal validity indices such as average silhouette width, Normalized Gamma statistics, Dunn’s index and separation index [68, 69] to evaluate quality of clustering results across *k* ∈ [2,|A| − 1].

We then summarized the results by calculating combined normalized ranks across these cluster quality indices by the following formula,

$$\text{score}\left(k=k^{\prime }\right)=\sum_{m^{\prime }\in M}\log\left(\frac{\text{rank}\left(k=k^{\prime },m=m^{\prime }\right)}{\left(\left|\Phi \right|-1\right)}\right)$$ (7)

where *M* is the set of cluster quality indices, rank(*k*, *m*) is the rank of k by the cluster quality index *m*, and score(*k*) is the summarized score of *k*.

**Fig 14:**
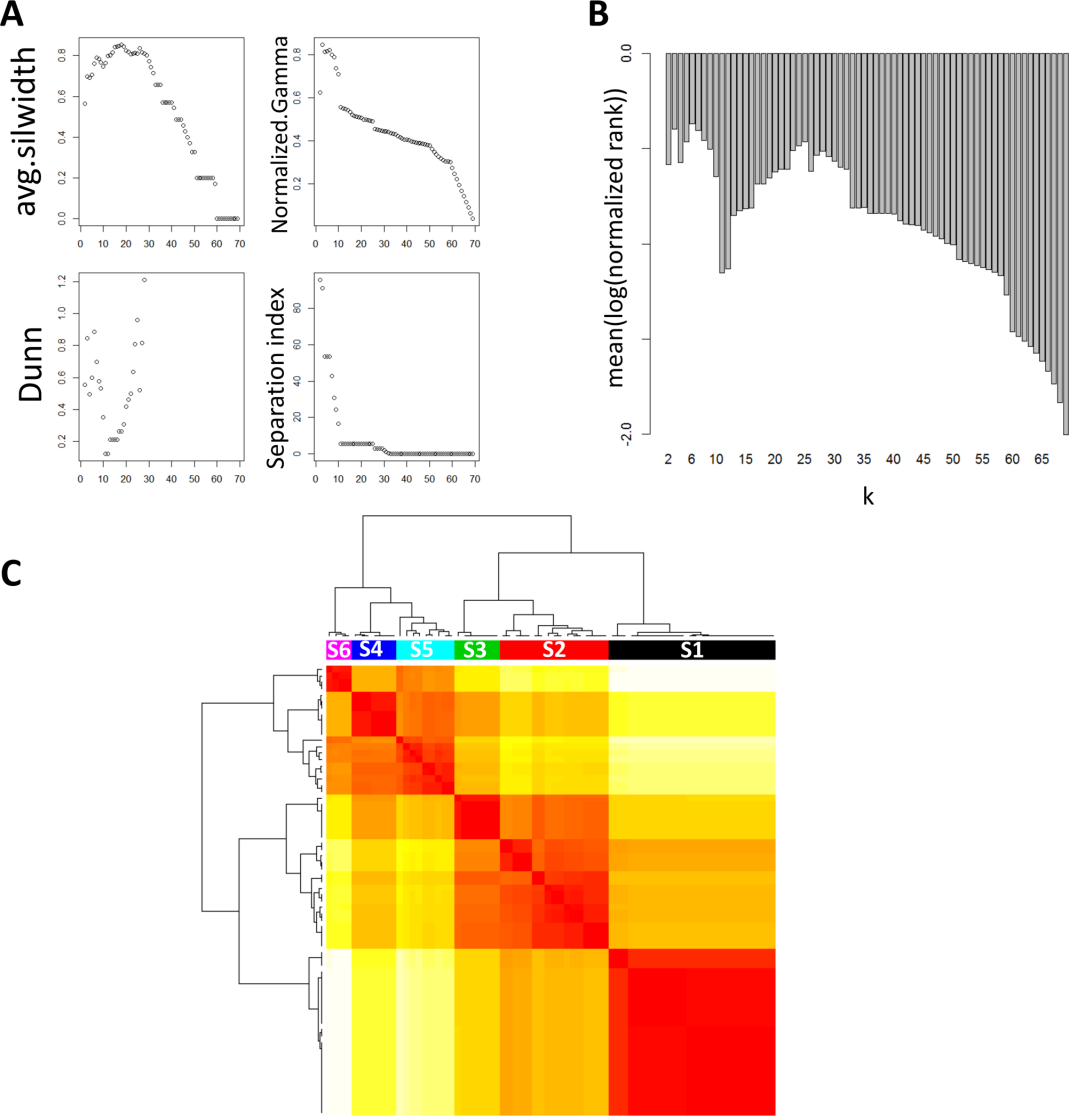
Identification of hubs at various scale (defined by α) groups in the breast cancer PFN. A) Plots of various internal validity indices used for selecting the optimal number of clusters to group α values. B) Barplot showing summarized scores from normalized ranks by internal validity indices from A). C) A heatmap of the pairwise Euclidean distances between any two vectors of the within-cluster connectivity (determined by *C* ^*w*^ *(V*,*A))* of all the nodes at the corresponding scales. The color bar on the top of heatmap represents the distinct scale clusters identified by MHA.

**Fig 14** shows the grouping process in analyzing the BRCA PFN where 6 distinct scales were identified.

#### Identification of hubs at each scale

For each subnetwork at a scale α, *n*_*s*_ random networks are generated as described previously in MCA description. The significance of within-cluster connectivity c^w^(*ν*_*i*_,*α*) is then evaluated by taking the average number of nodes that have higher connectivity than c^w^(*ν*_*i*_,*α*) from *n*_*s*_ random networks with *n*_*s*_ = 100 as default.

#### Identification of multiscale hubs

We adopt Fisher’s inverse Chi-square approach to compute the combined statistics for each node *i* across α values grouped together, termed a scale group. That is,

$$S_{i}=\sum_{\alpha \in A_{l}}-\log_{10}p_{i}^{\left(\alpha \right)}$$ (8)

where, $p_{i}^{\left(\alpha \right)}$ is significance p-value of c^w^(*ν*_*i*_,*α*), and *A*_*l*_ is the scale group including the set of α values grouped together by clustering for C^*w*^(*V*,A). In order to evaluate the significance of S_i_, we generate the null distribution of *S*_*i*_ by randomly shuffling for α and *i* in the equation above. This shuffling is performed N_s_ times to generate stable null distribution, where *N*_*s*_ = 100 by default. Nominal p-values for each of *S*_*i*_ is calculated from the null distribution, and is corrected for multiple testing by Bonferroni correction for the number of nodes in the PFN. We have set Bonferroni corrected p-value < 0.05 as the default threshold to identify significant hubs for each scale group *A*_*l*_.

### Cluster-Trait Association Analysis (CTA)

To relate each cluster with clinical outcomes, principal component analysis (PCA) is first performed for each cluster and then the correlation between the first (or multiple) principal component(s) and each trait is computed as cluster relevance to the trait.

For patient survival data, the association is examined by multivariate Cox proportional hazards regression model that regresses patient survival onto the first (or multiple) principal component(s) of a given module, and Cox p-value is calculated to evaluate the significance. To further investigate the prognostic power of each cluster, logrank p-value is calculated to characterize the difference between the survival curves of two molecular subtypes defined by the median expression of the first PC of each cluster. The logrank p-values and Cox p-values are then corrected for multiple testing by Benjamini–Hochberg FDR correction.

### Computational Complexity Analysis

Among the four major steps of MEGENA including FPFNC, MCA, MHA and CTA, FPFNC is most time consuming. The complexity of the existing serial PMFG algorithm has a complexity of O(|V|^γ^), 2 ≤ γ ≤ 3, where the worst case of O(|V|^3^) is due to performing O(|V|) Myrvold-Boyer planarity test on O(|V|^2^) correlation pairs. FPFNC circumvents this problem by reducing the number of correlation pairs subject to the planarity test by means of testing significance of every correlation pair, taking O(|V|^2^). Assuming that a certain threshold such as FDR < 0.05 leaves a faction of nodes correlation to every node, we can approximate the number of remaining pairs to construct PFN as ϵ|V|. Combining these two, the overall complexity is O(ϵ'|V|^2^), which is a substantial improvement over the previous algorithm. Furthermore, the parallelization via PCP with several cores allows to handle for the multiplicative factor ϵ', leading to O(ϵ''|V|^2^) with ϵ' > ϵ'' ≥ 1. As a result, FPFNC achieves a scalable computation of PFN of |V| ~ 20,000 with moderate computational resources within a few days, while the exiting serial PMFG algorithm takes over a week to handle a network with |V| ~ 5000. For instance, using FPFNC on 16 cores (3.5 GHz Intel Ivy Bridge), the LUSC PFN with |V| = 20523 (**Fig 2A and 2B**) was constructed in less than 36 hours and the THCA PFN with |V| = 16639 (**Fig 2C and 2D**) took less than 18 hours. Construction of such large-scale PFNs is not feasible for the existing serial PMFG algorithm as we estimate that it will take over a month.

MCA is governed by computation of shortest-path distance (SPD) for all pairs of nodes, and iterative k-medoids clustering. We adopted the Bellman-Ford algorithm to compute SPD[70] which has a computation complexity O(|V||E|). Given |E| ≤ 3(|V|-2) in embedded networks on surface with g = 0, the time complexity of computing SPD is O(|V|^2^). SPD is calculated for multiple times in MCA. It is calculated first from the global PFN as the input dissimilarity matrix for k-medoids clustering, and then from multiple random planar networks to calculate statistics for cluster compactness to evaluate each split. Therefore, the initial computation of SPD from PFN dominates the running time since computing SPDs for candidate clusters become relatively negligible due to dramatic decrease in cluster size. Therefore, the overall complexity involving all SPD calculations becomes O(ϵ|V|^2^), where ϵ corresponds to the number of clusters with sizes comparable to the PFN. Additionally, the computational complexity of k-medoids clustering is O(|V|^2^/k)[67], where k is tested from k = 2,…,k_max_ with k_max_ reaching around 50 in practical cases with current implementation of k-split. Therefore, the overall time complexity of MCA is O(ϵ'|V|^2^). In the current implementation, the overall computation time of MCA for a PFN with |V| = 15402 took less than 2 hours on a single core (3.5 GHz Intel Ivy Bridge).

Lastly, the computational complexity of MHA is dictated by calculation of significance of within-cluster connectivity for each node, across a range of α values. Given that we generate n_s_ (= 100 by default) random planar networks for each unique cluster, and we calculate degree of each node for each random network, the time complexity for performing the statistical test for each cluster is O(|V_l_|), and for all clusters is O(Σ_l_|V_l_|). Since Σ_l_|V_l_| ~ ϵ|V| where ϵ is the mean number of instances that a single node appears in different clusters, the overall time complexity for MHA is fairly linear with |V|. Indeed, MHA for the BRCA and LUAD PFNs in this manuscript took only few minutes.

Overall, the computation complexity of MEGENA is O(β|V|^2^), where β largely depends on the number of cores to perform parallelized computations. The space (memory) complexity of MEGENA is O(|V|^2^) due to a |V|x|V| similarity matrix. Based upon 16 cores of 3.5 GHz Intel Ivy Bridge, FPFNC just needed less than 3 hours to construct the BRCA and LUAD PFNs with 6999 and 7562 nodes, respectively while the existing PMFG algorithm took over a week. The whole MEGENA took less than 4 hours for both cases.

## Supporting Information

S1 Data Supporting data including the results from comparing Molecular Signature DataBase (MSigDB) gene sets from Gene Ontology (GO) collection and pathway databases (KEGG and REACTOME) via Fisher Exact Test (FET), testing for over-representation (i.e. odds ratio > 0). (TXT)

S2 Data Supporting data including the results from comparing Molecular Signature DataBase (MSigDB) gene sets from Gene Ontology (GO) collection and pathway databases (KEGG and REACTOME) via Fisher Exact Test (FET), testing for under-representation (i.e. odds ratio < 0). (TXT)

S1 Text Supporting information describing data acquisition and quality control, the established clustering methods in comparison with MEGENA, and the results from applying MEGENA to the TCGA LUAD dataset. (DOCX)

S1 Table The functionally annotated gene clusters (FAGCs) specifically identified by MEGENA. (DOCX)

S1 Fig Comparison of Coefficient of Variation (CV) across various FDR thresholds per simulated data per network inference method. The error bars show standard deviation across 10 simulated data sets from a golden standard network from DREAM challenge.
(TIF)

S2 Fig A global PFN in LUAD. Node border colors represent different clusters identified at a scale α = 1. Node size and label size are proportional to node degree. Node fill colors are proportional to tumor expression fold changes in comparison to matched normal samples. Hub genes identified at any scale are labeled by gene symbols with font sizes proportional to the node degree.
(TIF)

S3 Fig Comparison of MEGENA (as a combination of the multiscale clustering analysis and PFN) and various combinations of the established clustering techniques (eigenvector, infomap, walktrap, WGCNA) and the networks (PFN, FDRN, WGCN) using the TCGA LUAD gene expression data. A) The number of significantly enriched functional/pathway signatures (Bonferroni corrected FET p-values) from MSigDB at various p-value thresholds. B) Number of significantly enriched functional/pathway signatures from MSigDB at the various odds ratio thresholds. C) Number of clusters predictive of patient survival (based on FDR corrected logrank p-values) at various significance levels.
(TIF)

S4 Fig Dependency of prognostic significance of cluster-defined molecular subgroups on cluster size in BRCA (A) and LUAD (B). Point shapes denote different clustering methods and point colors represent different co-expression networks, and point sizes represent significance of logrank p-values with FDR corrected p-value < 0.05 threshold.
(TIF)

S5 Fig Identification of the multiscale clustering organization in the LUAD PFN. A) Summarization scores from normalized ranks by various internal validity indices for clustering solutions across k. B) A heatmap of the pairwise Euclidean distances between α values. The distance was computed from the within-cluster connectivity matrix, Cw(V,A). The colorbar on top of the heatmap labels the scale clusters. C) The number of GO/KEGG/MSigDB gene sets enriched in the clusters at each scale group across a spectrum of Bonferroni corrected FET p-values.
(TIF)

S6 Fig Comparison of the distribution of the expression fold changes of the hub genes and that of the non-hub genes at each scale in the TCGA BRCA network. Each subplot compares two different stages of breast cancer. The y-axis represents–log10(Kolmogorov-Smirnov test p-value) and the x-axis represents different scales. The horizontal red line corresponds to KS p-value = 0.05.
(TIF)

S7 Fig Comparison of the expression fold changes (fc) of the hub genes and those of the non-hub-genes at each scale in the LUAD network. The x-axis shows the non-hub gene set and the hub gene sets at different scales. The category “multiscale” represents the hub gene set across all the different scales and the one “non.hub” represents the rest of genes. The y-axis shows the absolute values of log2(fc) between different cancer stages in LUAD.
(TIF)

S8 Fig Comparison of the distribution of the expression fold changes of the hub genes and that of the non-hub genes at each scale in the TCGA LUAD network. Each subplot compares two different stages of breast cancer. The y-axis represents–log10(Kolmogorov-Smirnov test p-value) and the x-axis represents different scales. The horizontal red line corresponds to KS p-value = 0.05.
(TIF)

S9 Fig Enrichment of the knock-down signatures of GPR116 (upper panel) and HOPX (lower panel) in the neighborhoods of the corresponding targeted genes in the LUAD PFN. Red horizontal lines show Bonferroni corrected FET p-value = 0.05 for the number of nodes in LUAD PFN.
(TIF)
