# Supplementary material for: Multiscale Embedded Gene Co-expression Network Analysis
Source: PLoS Comput Biol. 2015 Nov 30;11(11):e1004574. doi: 10.1371/journal.pcbi.1004574 (PMC4664553; doi:10.1371/journal.pcbi.1004574)
Supplement: S1 Text — (DOCX) [file pcbi.1004574.s003.docx]

**Supporting Text S1**

**Data acquisition and quality control**

The RNASeqV2 based gene expression data (Combination of MapSplice and RSEM to determine expression level) from the TCGA database (<https://tcga-data.nci.nih.gov/tcga/findArchives.htm>) were first log-transformed, then quantile normalized and finally corrected for the known cofounding factors including batch, tissue source site, center, plate, race, gender and age.

Based on the processed data, the genes accounting for 80% of total variance were selected for the network construction, leading to 6999 genes across 307 tumor samples for lung adenocarcinoma data and 7526 genes across 844 tumors.

**Execution of established clustering methods on BRCA and LUAD data**

WGCNA

The weighted network analysis begins with a matrix of the similarities such as Pearson correlations between all gene pairs, then converts the correlation matrix into an adjacency matrix using a power function *f(x)=x^β^*. The parameter *β* of the power function is determined in such a way that the resulting adjacency matrix, i.e., the weighted co-expression network, is approximately scale-free. To measure how well a network satisfies a scale-free topology, we use the fitting index, i.e., the model fitting index *R^2^* of the linear model that regresses *log(p(k))* on *log(k),* where *k* is connectivity and *p(k)* is the frequency distribution of connectivity[^1^](#_ENREF_1). The fitting index of a perfect scale-free network is 1. The connectivity between genes or *k_ij_* is a transformed correlation between the expression profiles of two genes, *|r(i,j)|^β^*, with *r* as the similarity. The parameter *β (>0)* of the power function is determined in such a way that the global probability distribution of the resulted connectivity values for all the gene pairs is scale free.

To explore the modular structures of a co-expression network, we further transform the corresponding adjacency matrix into a topological overlap matrix (TOM) and then employ average linkage hierarchical clustering to group genes based on the topological overlap of their connectivity, followed by a dynamic cut-tree algorithm to dynamically cut clustering dendrogram branches into gene modules [^2^](#_ENREF_2). To distinguish between modules, each module is assigned a unique color identifier, with the remaining, less well connected genes colored grey.

Informap, walktrap and leading eigenvector based spectral clustering

The established clustering techniques including Informap, walktrap and leading eigenvector based spectral clustering, were compared with the multiscale clustering analysis. These methods were implemented in the “igraph” R package[^3^](#_ENREF_3).

**Application to the TCGA LUAD data**

Comparison of different clustering techniques

We have compared MEGENA to a number of clustering techniques in PFN and FDRN for LUAD data. **S1** **Figure** shows the resulting LUAD PFN at α=1. Similar to the case of the network analysis of the BRCA data, the clusters from various combinations of the clustering techniques and network construction approaches were tested for enrichment of the gene sets in GO/KEGG/MSigDB databases, and for association with overall survival. **S2 Figure A** shows that the clusters of MEGENA, i.e., multiscale clustering on PFN, are enriched for the most number of the annotated functional gene sets at various thresholds for enrichment test p-value. Furthermore, multiscale clustering identifies clusters across wide range of sizes that are significantly associated to survival outcomes with FDR corrected logrank p-value < 0.05 in **S2 Figure B**. This pattern is consistently observed in **S2 Figure C** where gene expression clusters were used to identify subgroups with significant differences in overall survival outcomes.

MHA of LUAD network

MHA identifies two distinctive groups of scales are present in the LUAD global PFN as shown in **S5 Figure**. Similarly to the case study of BRCA, we evaluated biological relevance of the multiscale hubs from MHA in the context of LUAD progression by comparing expression fold changes between different cancer stages. **S7 Figure** shows that the hub genes as a whole have increased expression fold change in comparison to the non-hub genes and **S8 Figure** shows the significance of the difference in each comparison at each scale by the Kolmogorov-Smirnov test. These results are consistent with what we found from the case study of BRCA.

**References**

1 Zhang, B. & Horvath, S. A general framework for weighted gene co-expression network analysis. *Stat Appl Genet Mol Biol* **4**, 12 (2005).

2 Langfelder, P., Zhang, B. & Horvath, S. Defining clusters from a hierarchical cluster tree: the Dynamic Tree Cut package for R. *Bioinformatics* **24**, 719-720 (2008).

3 Csardi, G. & Nepusz, T. The igraph software package for complex network research. *InterJournal* **Complex Systems**, 1695 (2006).
