# Supplementary figures and images for: Multiscale Embedded Gene Co-expression Network Analysis
Source: PLoS Comput Biol. 2015 Nov 30;11(11):e1004574. doi: 10.1371/journal.pcbi.1004574 (PMC4664553; doi:10.1371/journal.pcbi.1004574)

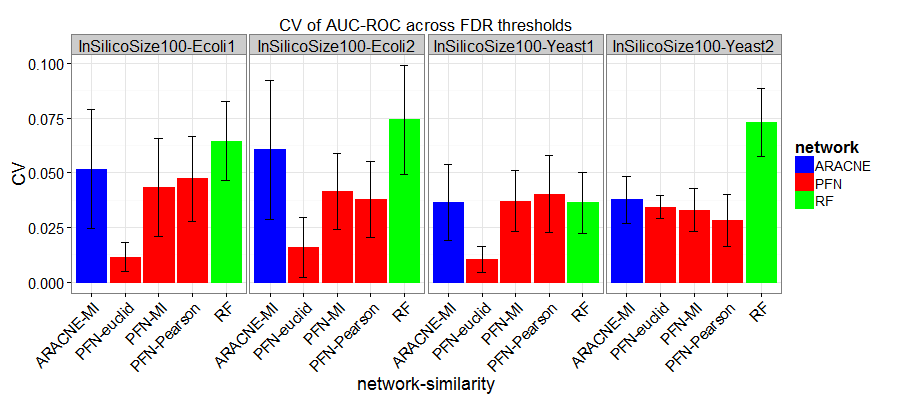

Supplement: S1 Fig — The error bars show standard deviation across 10 simulated data sets from a golden standard network from DREAM challenge. (TIF) [file pcbi.1004574.s005.TIF]

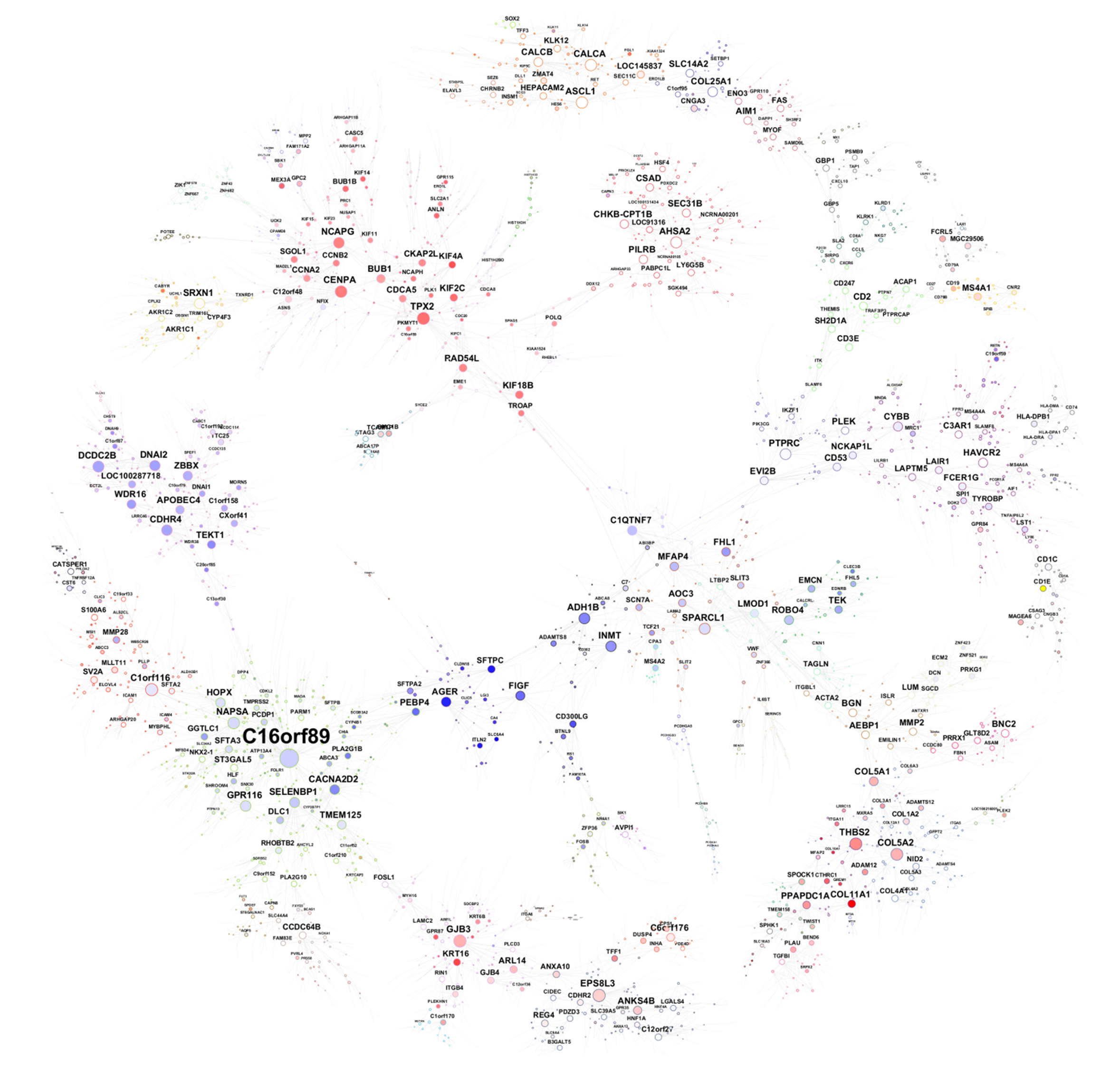

Supplement: S2 Fig — Node border colors represent different clusters identified at a scale α = 1. Node size and label size are proportional to node degree. Node fill colors are proportional to tumor expression fold changes in comparison to matched normal samples. Hub genes identified at any scale are labeled by gene symbols with font sizes proportional to the node degree. (TIF) [file pcbi.1004574.s006.TIF]

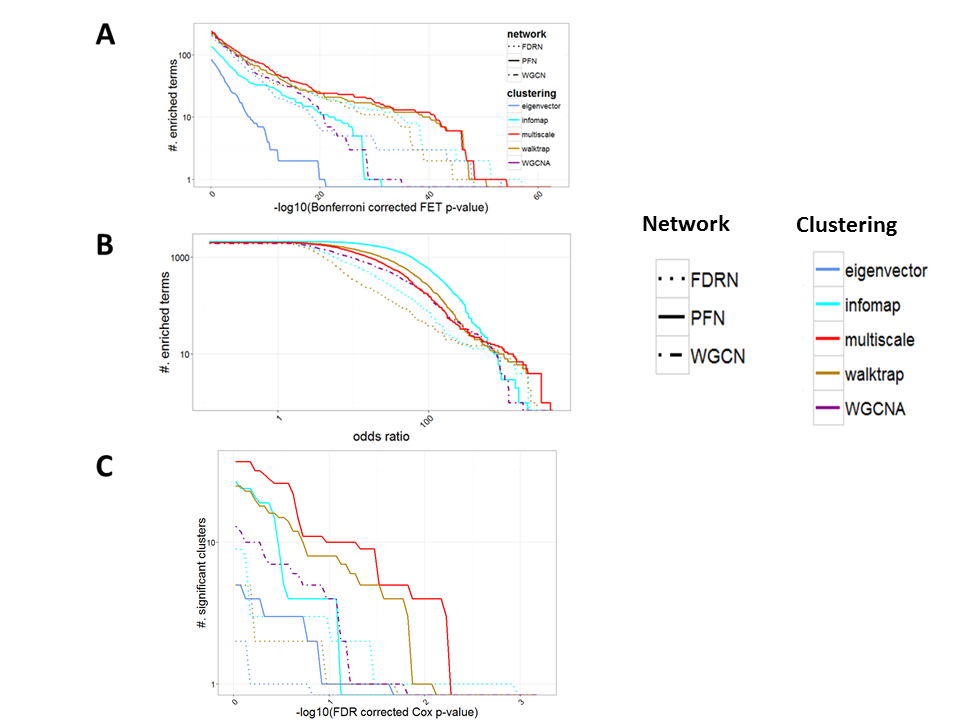

Supplement: S3 Fig — A) The number of significantly enriched functional/pathway signatures (Bonferroni corrected FET p-values) from MSigDB at various p-value thresholds. B) Number of significantly enriched functional/pathway signatures from MSigDB at the various odds ratio thresholds. C) Number of clusters predictive of patient survival (based on FDR corrected logrank p-values) at various significance levels. (TIF) [file pcbi.1004574.s007.TIF]

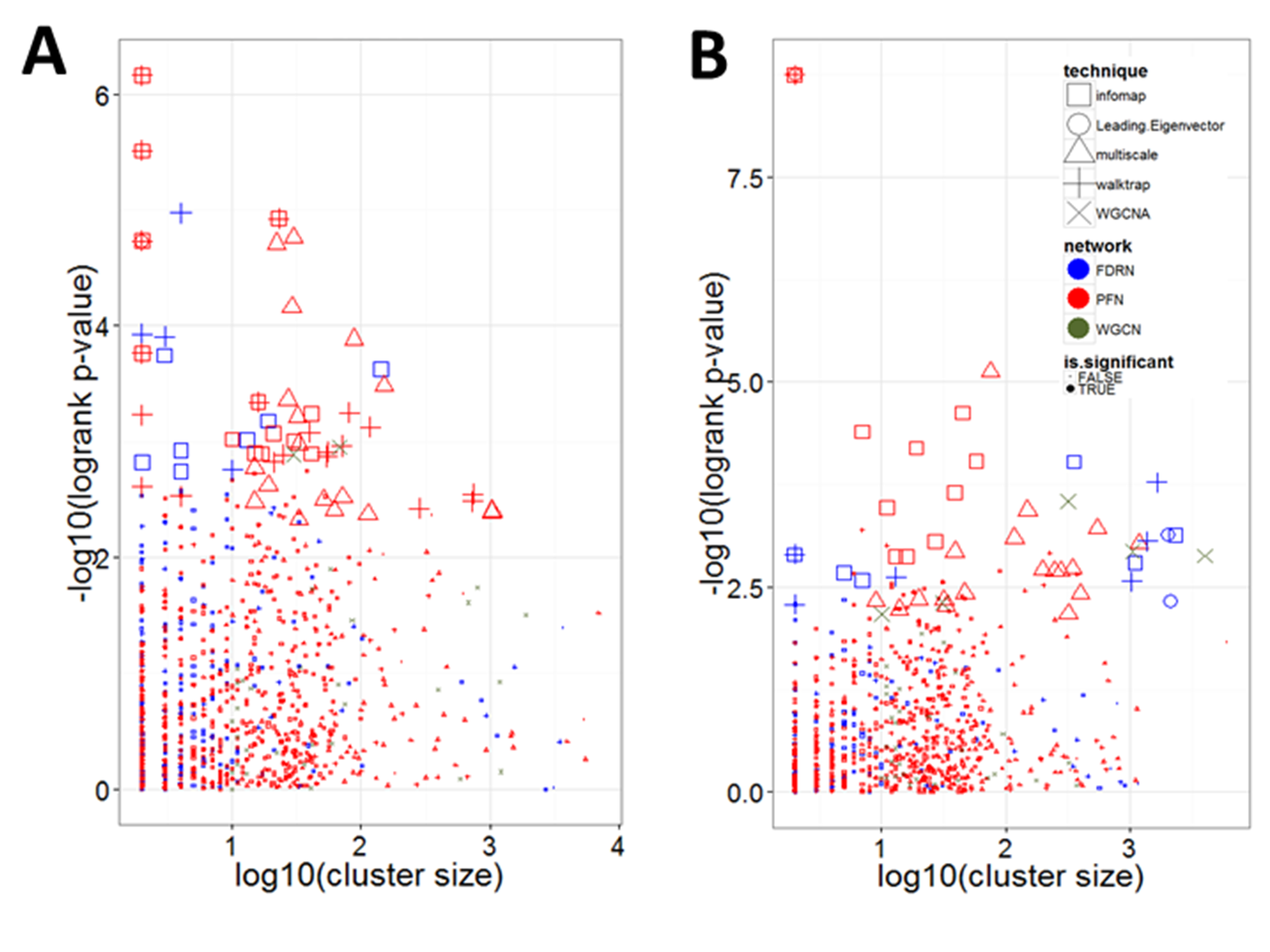

Supplement: S4 Fig — Point shapes denote different clustering methods and point colors represent different co-expression networks, and point sizes represent significance of logrank p-values with FDR corrected p-value < 0.05 threshold. (TIF) [file pcbi.1004574.s008.TIF]

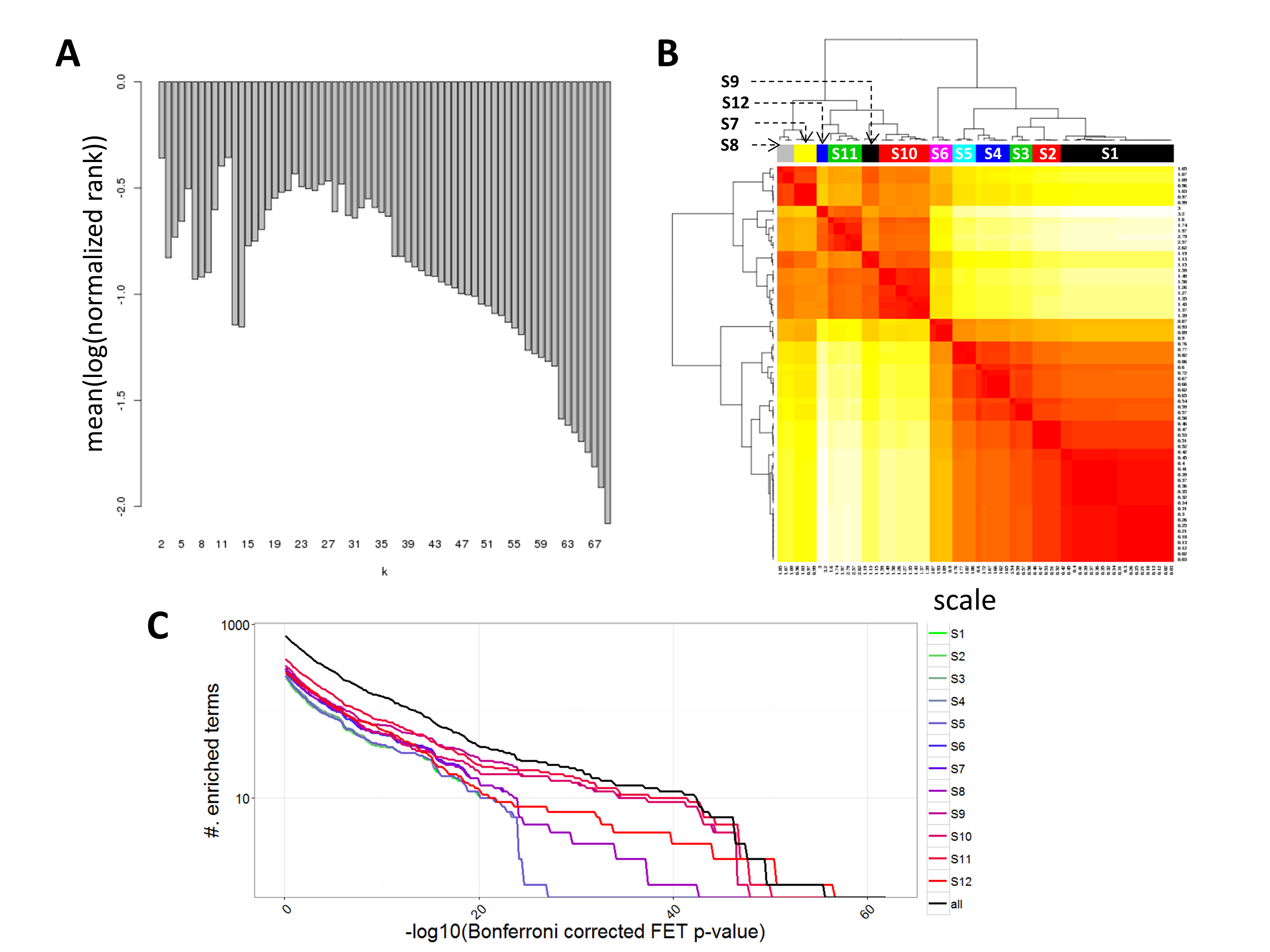

Supplement: S5 Fig — A) Summarization scores from normalized ranks by various internal validity indices for clustering solutions across k. B) A heatmap of the pairwise Euclidean distances between α values. The distance was computed from the within-cluster connectivity matrix, Cw(V,A). The colorbar on top of the heatmap labels the scale clusters. C) The number of GO/KEGG/MSigDB gene sets enriched in the clusters at each scale group across a spectrum of Bonferroni corrected FET p-values. (TIF) [file pcbi.1004574.s009.TIF]

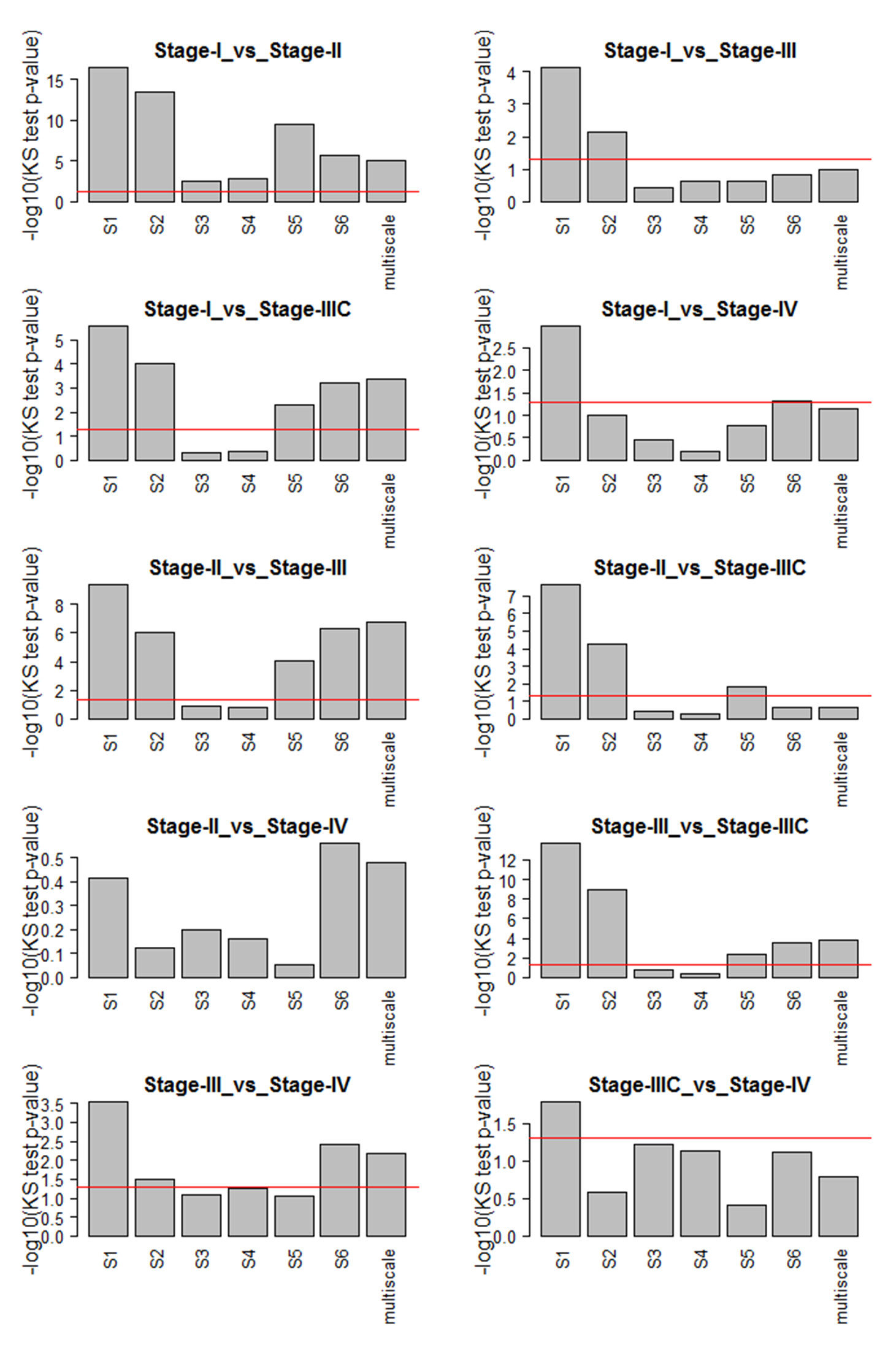

Supplement: S6 Fig — Each subplot compares two different stages of breast cancer. The y-axis represents–log10(Kolmogorov-Smirnov test p-value) and the x-axis represents different scales. The horizontal red line corresponds to KS p-value = 0.05. (TIF) [file pcbi.1004574.s010.TIF]

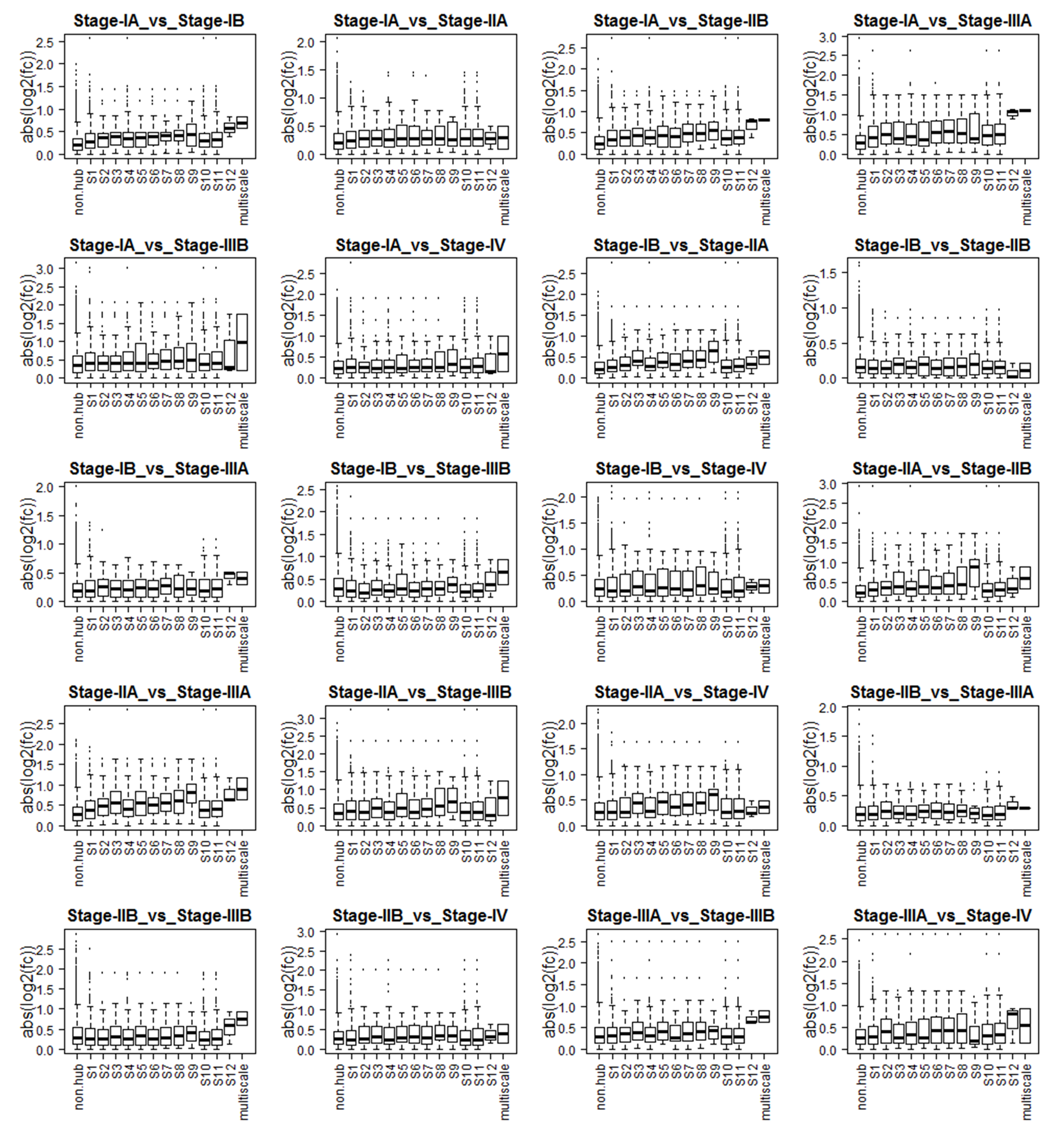

Supplement: S7 Fig — The x-axis shows the non-hub gene set and the hub gene sets at different scales. The category “multiscale” represents the hub gene set across all the different scales and the one “non.hub” represents the rest of genes. The y-axis shows the absolute values of log2(fc) between different cancer stages in LUAD. (TIF) [file pcbi.1004574.s011.TIF]

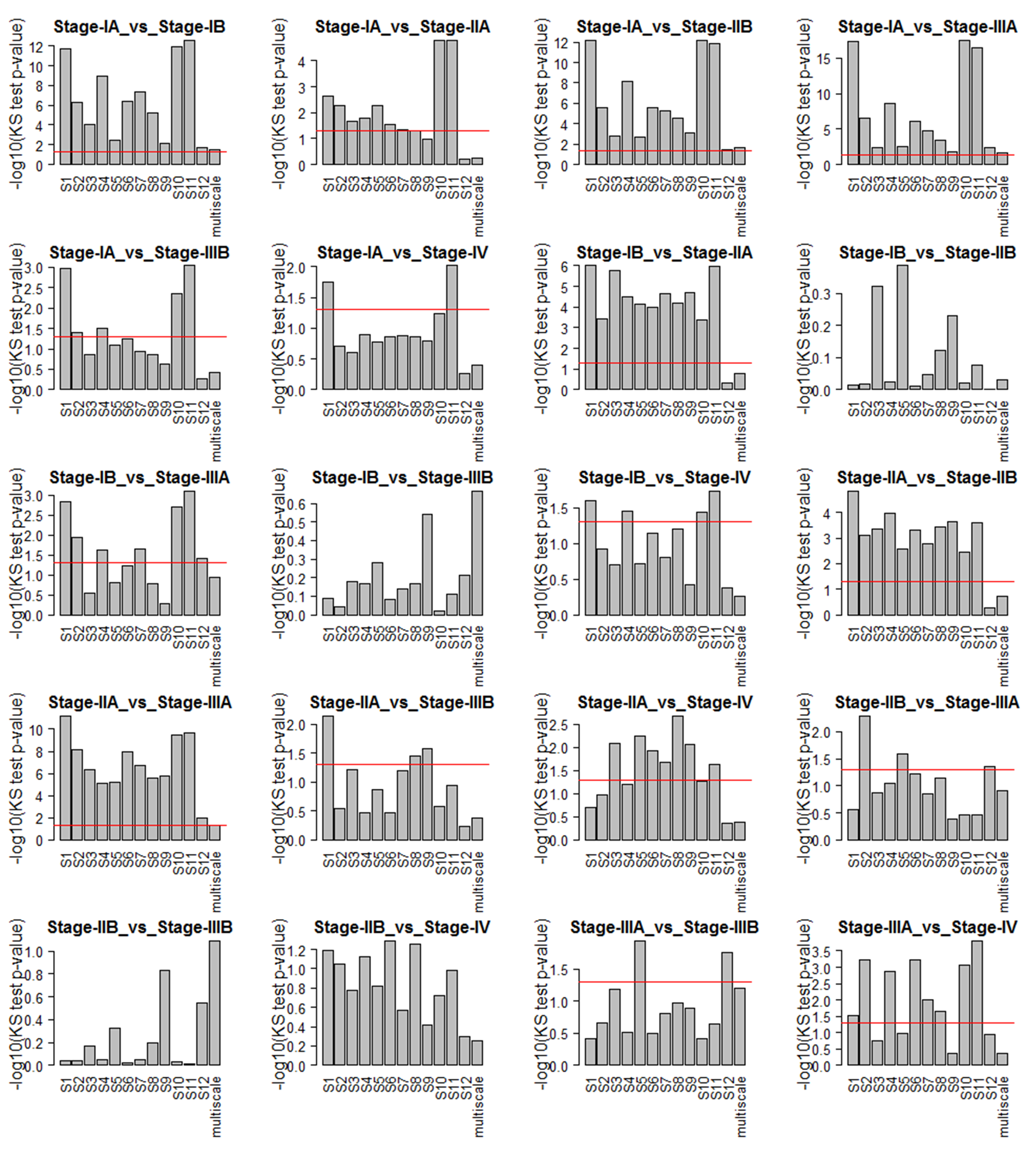

Supplement: S8 Fig — Each subplot compares two different stages of breast cancer. The y-axis represents–log10(Kolmogorov-Smirnov test p-value) and the x-axis represents different scales. The horizontal red line corresponds to KS p-value = 0.05. (TIF) [file pcbi.1004574.s012.TIF]

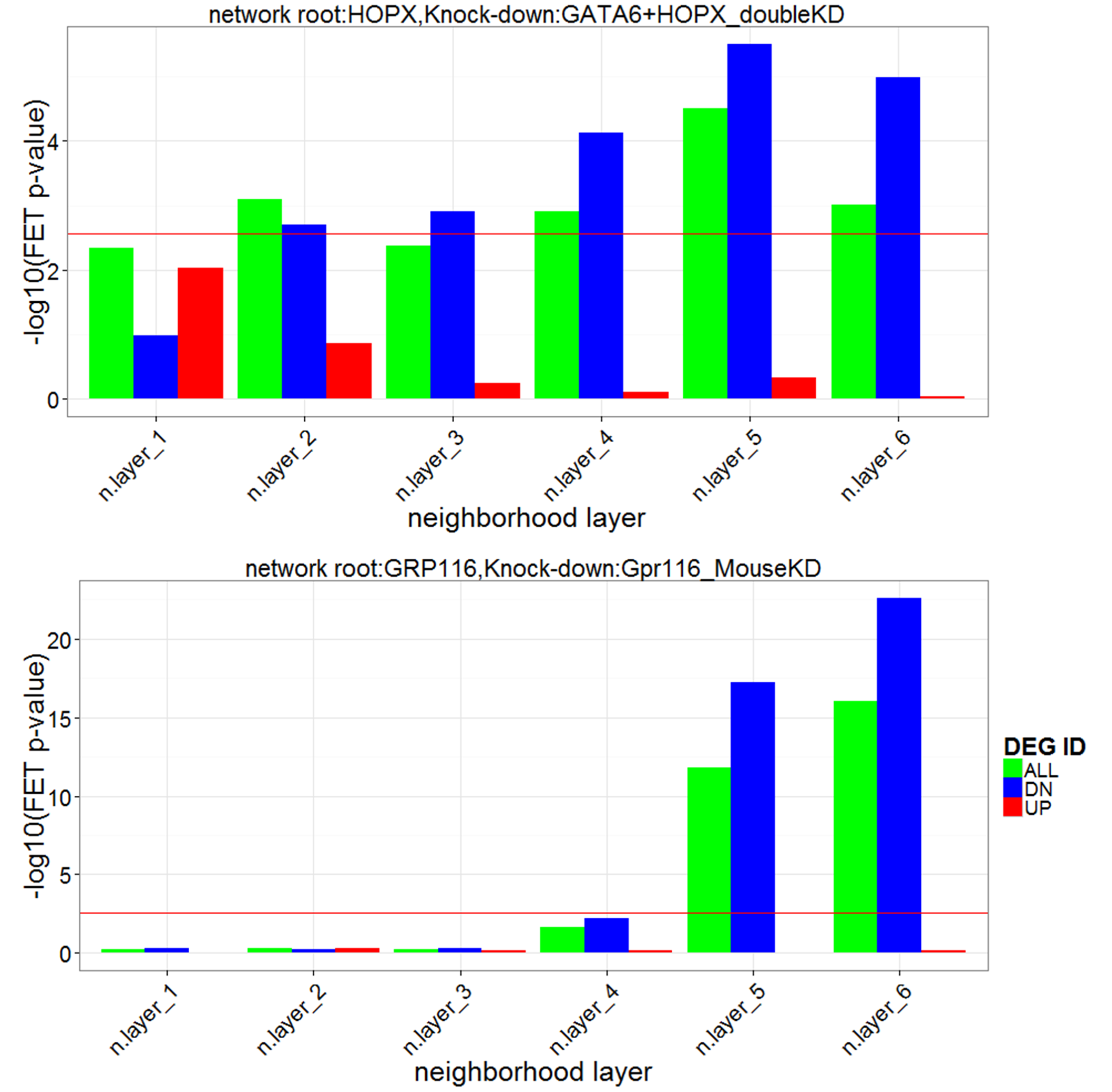

Supplement: S9 Fig — Red horizontal lines show Bonferroni corrected FET p-value = 0.05 for the number of nodes in LUAD PFN. (TIF) [file pcbi.1004574.s013.TIF]
