# Supplementary material for: Multiscale Embedded Gene Co-expression Network Analysis
Source: PLoS Comput Biol. 2015 Nov 30;11(11):e1004574. doi: 10.1371/journal.pcbi.1004574 (PMC4664553; doi:10.1371/journal.pcbi.1004574)
Supplement: Supplementary file 5 [file pcbi.1004574.s004.docx]

Database	Cluster	Category	Cluster Size	Category Size	Overlap	Fold Enrichment	FET p-value	Corrected FET p-value
GO-BP	comp1_4	CELLULAR_HOMEOSTASIS	931	91	30	2.664982708	2.09E-07	0.02488244
GO-BP	comp1_4	CELL_PROLIFERATION_GO_0008283	931	237	57	1.944200465	3.47E-07	0.041230819
GO-BP	comp1_4	CHEMICAL_HOMEOSTASIS	931	96	31	2.610387576	2.23E-07	0.026507067
KEGG	comp1_10	KEGG_ADIPOCYTOKINE_SIGNALING_PATHWAY	105	27	6	15.92804233	1.49E-06	0.039927811
KEGG	comp1_12	KEGG_LEUKOCYTE_TRANSENDOTHELIAL_MIGRATION	437	54	14	4.464954657	1.53E-06	0.040995587
KEGG	comp1_33	KEGG_PROGESTERONE_MEDIATED_OOCYTE_MATURATION	463	34	12	5.737009275	4.03E-07	0.010798775
KEGG	comp1_56	KEGG_ADIPOCYTOKINE_SIGNALING_PATHWAY	100	27	6	16.72444444	1.12E-06	0.02993356
REACTOME	comp1_4	REACTOME_IL_3_5_AND_GM_CSF_SIGNALING	931	14	10	5.774129201	5.02E-07	0.04875052
REACTOME	comp1_7	REACTOME_SIGNALING_BY_PDGF	536	41	14	4.794503094	4.27E-07	0.041426334
REACTOME	comp1_30	REACTOME_TRANSMISSION_ACROSS_CHEMICAL_SYNAPSES	149	113	13	5.810892677	2.98E-07	0.028880066
REACTOME	comp1_41	REACTOME_SIGNALING_BY_PDGF	318	41	12	6.926829268	6.90E-08	0.006695092
REACTOME	comp1_102	REACTOME_SIGNALING_BY_PDGF	244	41	11	8.275289884	4.44E-08	0.004309316
REACTOME	comp1_102	REACTOME_NCAM1_INTERACTIONS	244	31	9	8.954785828	3.69E-07	0.035812595
